# Performance efficient macromolecular mechanics via sub-nanometer shape based coarse graining

**DOI:** 10.1038/s41467-023-37801-5

**Published:** 2023-04-10

**Authors:** Alexander J. Bryer, Juan S. Rey, Juan R. Perilla

**Affiliations:** Department of Chemistry and Biochemistry, University of Delaware, Newark, DE 19716 USA

**Keywords:** Computational biophysics, Molecular dynamics, Software, Computational models, Molecular modelling

## Abstract

Dimensionality reduction via coarse grain modeling is a valuable tool in biomolecular research. For large assemblies, ultra coarse models are often knowledge-based, relying on a priori information to parameterize models thus hindering general predictive capability. Here, we present substantial advances to the shape based coarse graining (SBCG) method, which we refer to as SBCG2. SBCG2 utilizes a revitalized formulation of the topology representing network which makes high-granularity modeling possible, preserving atomistic details that maintain assembly characteristics. Further, we present a method of granularity selection based on charge density Fourier Shell Correlation and have additionally developed a refinement method to optimize, adjust and validate high-granularity models. We demonstrate our approach with the conical HIV-1 capsid and heteromultimeric cofilin-2 bound actin filaments. Our approach is available in the Visual Molecular Dynamics (VMD) software suite, and employs a CHARMM-compatible Hamiltonian that enables high-performance simulation in the GPU-resident NAMD3 molecular dynamics engine.

## Abstract (web summary)

Here the authors report SBCG2 an update to the neural network based, Shape-Based Coarse Graining (SBCG) approach for creating coarse grained molecular topologies with atomistic detail. They show how SBCG2 can reduce the computational costs of simulating very large assemblies like the HIV-1 capsid allowing simulation on commodity hardware.

## Introduction

Molecular dynamics (MD) simulations evolve chemical systems over time via integration of Newton’s equations of motion^1^. Since its inception as an investigatory method, MD simulation has provided high spatial and temporal resolution data of materials, surfaces, and biomolecular systems that complement experimentally derived information. A widely-recognized challenge in the domains of computational biochemistry and physics, which has driven the development of novel hardware^2^ and software alike, is the computational complexity of biomolecular systems.

As early as 1975, dimensionally-reduced descriptions of molecular systems have been employed to lessen the computational cost associated with protein folding simulation^3^. This practice, referred to generally as coarse-graining (CG), produces models that seek to accurately represent chemical systems with far fewer degrees of freedom than the 3(*N*_atoms_ − 1) present at atomistic resolution (with periodic boundary conditions)^4^. The scope and strategy of CG simulations have been revolutionized many times over in the last ~50 years and CG modeling has been successfully applied to gas, liquid, and condensed phase systems^5–10^. While the geometric increase in computing power of the late 20th century until the present has made atomistic simulation more computationally tractable, CG modeling has remained a staple of computational science. Considering our growing understanding of climate change and the extreme energy costs of supercomputing, the latter of which continues to balloon with ever-increasing computing power, we anticipate that dimensionality reduction via CG modeling will remain a staple for decades to come.

In general, coarse-graining refers to mapping, by various criteria, groups of atoms in ${{\mathbb{R}}}^{3}$ to a single position, or bead. In the present context, the term granularity refers to the degree of reduction, i.e., the coarseness of a given model, or how many atoms are mapped to a single bead. Granularity depends on several factors and is, in general, established by scientists based on the nature of their system and the questions they seek to investigate.

On the high-granularity end of the spectrum, MARTINI^11–13^ is a popular offering. Parameterized empirically, MARTINI maps four atoms to a single bead. The MARTINI force field contains bond, angle, dihedral, and nonbonded interaction terms that govern model behavior. On the low-granularity end of the spectrum, so-called ultra coarse-grained (UCG) models map many atoms, sometimes entire protein domains in biomolecular simulation, to a single bead^14^. Particularly adept at modeling large assemblies^15^ as well as processes of self-assembly^16–18^, UCG simulations commonly employ large integration time steps^19^ that increase sampling efficiency and aid the resolution of long timescale behavior. The flexibility and efficiency of low-granularity CG has enabled the study of flagellar motility^20^, as well as large-scale DNA dynamics via alternate levels of theory such as worm-like chain modeling^21^. Resulting from significant information loss due to extreme coarseness and low-granularity, UCG models are often knowledge-based; that is, the interactions among constituent beads are often established explicitly to reproduce known behavior in the absence of charge, hydrophobicity or other physical properties lost in the reduction. Multiscale CG models^22^ and force fields, e.g., SIRAH^23^, PACE^24,25^, and others, span the gap between low- and high-granularity regimes and have proven especially useful in CG descriptions of aqueous environments.

Construction and parameterization of CG models of varying granularity have also motivated the usage of machine learning (ML)^26–30^. The high dimensionality of molecular models, even coarse models, lends utility to neural networks suited for high dimensional optimization problems. The general paradigm of machine learning is to evolve a set of numerical weights, given a particular input pattern, in response to an objective function. Commonly, the optimization is supervised. That is, the objective function is supplied with training data, often experimental or high-resolution data, e.g., collected from atomistic simulation, to evaluate and evolve network parameters. This flexible approach has been successfully utilized in a variety of settings, including generalized and systematic CG force field optimization^26^ and CG modeling of water^27^. Derivation of CG force fields for dissipative particle dynamics (DPD) has also been accomplished with a Bayesian network^29^, another supervised scheme employing Bayesian inference for parameter optimization in the absence of a fully-realized objective function. A specific class of models, generative adversarial networks (GANs), employ competing prediction networks in a zero-sum game and learn to reproduce training data through optimization. GANs represent a semi- or weakly-supervised paradigm and have been successfully applied to the derivation of CG models^28^. Another hybrid architecture permitting weak supervision is the graph neural network (GNN), and this has also been productively utilized to optimize CG force fields^30^. Due to the dependence of supervised learning on robust, unbiased, and voluminous training data, unsupervised learning is an attractive strategy for a variety of problems.

A separate class of analytical methods for deriving CG potentials from atomistic data has also been developed. These frameworks, built on numerical optimization, have found significant utility at large scales. Approaches such as Lennard-Jones (LJ) static potential matching employ numerical optimization to define the LJ interaction potential of CG beads based on an atomistic force field^31^. Other techniques, such as force matching and Boltzmann inversion, have been successfully utilized to derive force field terms in numerous CG contexts, ranging from organic polymers^32^ to meso and multiscale biomolecular assemblies^22,32–34^. The relative entropy approach is an alternative optimization method that is additionally able to quantify the error of a CG model relative to a model built from the first principles^35^. Similar to unsupervised learning methods, analytical approaches to deriving CG potentials have low dependence on high-resolution data compared to supervised learning via, e.g., a convolutional neural network. These methods are well-suited for the efficient parameterization of macromolecular complexes.

The criteria that a CG reduction employs to yield models with particular granularities are often defined empirically. For example, a scientist who aims to simulate a viral capsid, encompassing tens of millions of atoms, may elect to employ a CG approximation, and then establish only one or two CG beads per capsid subunit based on information such as the presence of independently-folded protein domains and experimentally derived structures^36,37^. While serviceable to the foundational goals of the CG effort, such models suffer from model-dependent realism, and thus their predictive capabilities are contextually limited.

A desirable quality of CG modeling is transferability, i.e., the readiness of the CG reduction to be applied to other structures and systems while retaining general predictive potential^31,38^. To satisfy this requirement, the CG reduction should be algorithmic and thus predictable. Further, the reduction should retain enough information about the atomistic input to faithfully reproduce its natural properties, such as multimeric assembly characteristics, without the need for explicit parameterization.

In response to these needs, we present a significant revitalization to the formulation and implementation of the legacy shape-based coarse-graining (SBCG) approach^39–43^—introduced more than a decade ago—resulting in a state-of-the-art SBCG methodology that is highly transferable, and faithfully reproduces the atomistic behavior of large biomolecular assemblies. Specifically, in contrast to the legacy SBCG implementation: (1) we introduced conditions to the neural network that overcome hard limits intrinsic to SBCG granularity; (2) we developed a metric to establish the effective resolution of a particular granularity selection; (3) we developed a systematic iterative refinement of coarse-grained bond and angle parameters; and (4) we established a methodology to determine the parameters of the integrator to perform SBCG molecular dynamics simulations in modern computing architectures including graphical processing units (GPUs). Overall, we refer to the next-generation coarse-graining methodology as SBCG2. In the following sections, we describe SBCG2 in detail, showing a methodology that enables SBCG2 to access high-granularity regimes, and that resulting models conform to atomistic charge density profiles within sub-nanometer resolution. As mentioned before, we describe the derivation of a methodology for bond and angle parameter optimization via iterative Boltzmann inversion and discuss considerations for its deployment in high-granularity, sub-nanometer use-cases. We demonstrate the utility of the SBCG2 method via application to three unique protein structures, comprising two macromolecular biological assemblies: human cofilin-2 bound to actin filaments^44^ and the full-scale HIV-1 conical capsid. In addition, we show the use of SBCG2 to probe the mechanoelastic properties of the HIV-1 capsid.

## Results

**Fig. 1:**
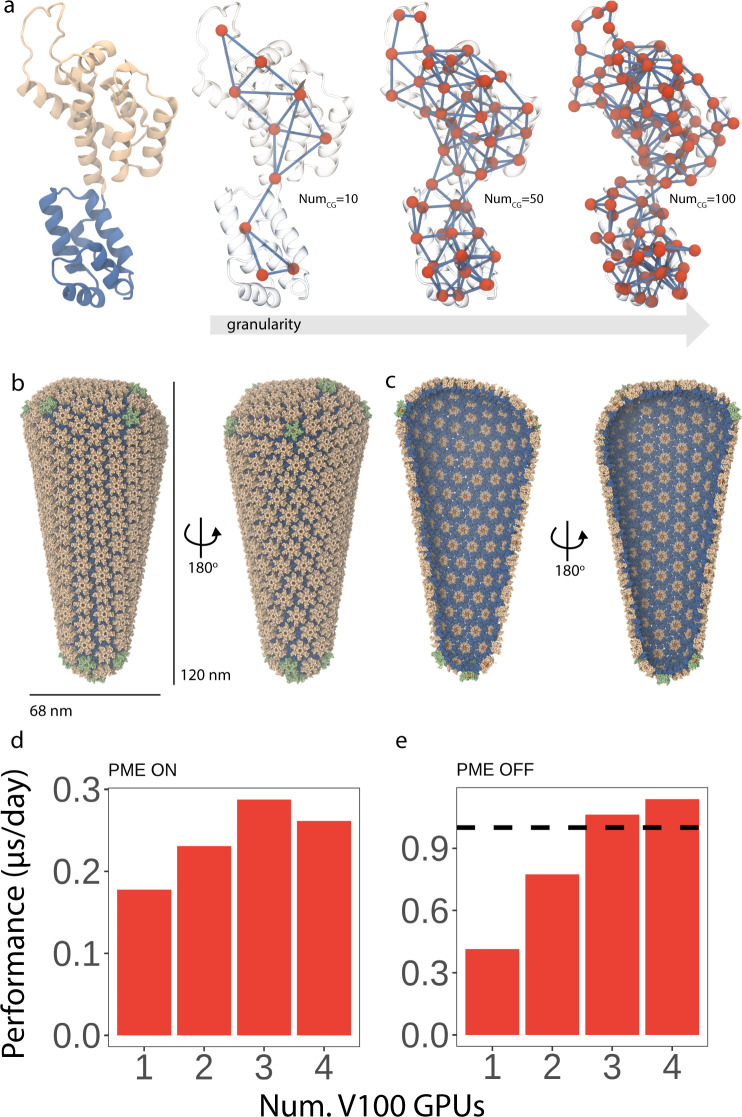
Shape-based coarse-grained 2 (SBCG2) HIV-1 capsid. **a** View of the HIV-1 CA monomer, left, colored by domain. On the right, three corresponding SBCG2 models of HIV-1 CA with increasing granularity. Granularity, in this case, refers to the number of beads employed to model the structure. **b** Full view of the SBCG2 HIV-1 conical capsid, shown from two perspectives. **c** Clipped view of the SBCG2 HIV-1 conical capsid, shown from two perspectives. For panels **b** and **c**, protein is shown as vdW beads, with the CA amino-terminal domains colored tan and the CA carboxy-terminal domains colored blue. IP6 beads are shown as orange vdW spheres^46,47^. **d**, **e** Performance benchmarks with NAMD3^45^, simulating the HIV-1 conical capsid shown in panels **c** and **d**. Benchmarks were performed with one CPU per GPU employed. For both benchmarks, an identical configuration was employed, and only the usage of PME for long-range electrostatics varied. The time step employed was 48 fs per step; Langevin *γ* was set to 2.0 ps^−1^; bonded interactions were evaluated every time step and nonbonded interactions were evaluated every other time step. **d** NAMD3 GPU benchmarks, utilizing NVIDIA V100s, with PME *on*. Peak performance of nearly 300 ns per day represents a threefold speedup over peak CPU-only simulation performance, which employed as many as ten compute nodes (Supplementary Fig. 2). **e** NAMD3 GPU benchmarks, utilizing NVIDIA V100s, with PME off. Remarkably, for three and four GPUs per simulation, we exceed one microsecond per day simulation performance (dashed line). Benchmarks reported are the mean value of the six benchmark metrics reported by NAMD3^45^ for each simulation.

**Fig. 2:**
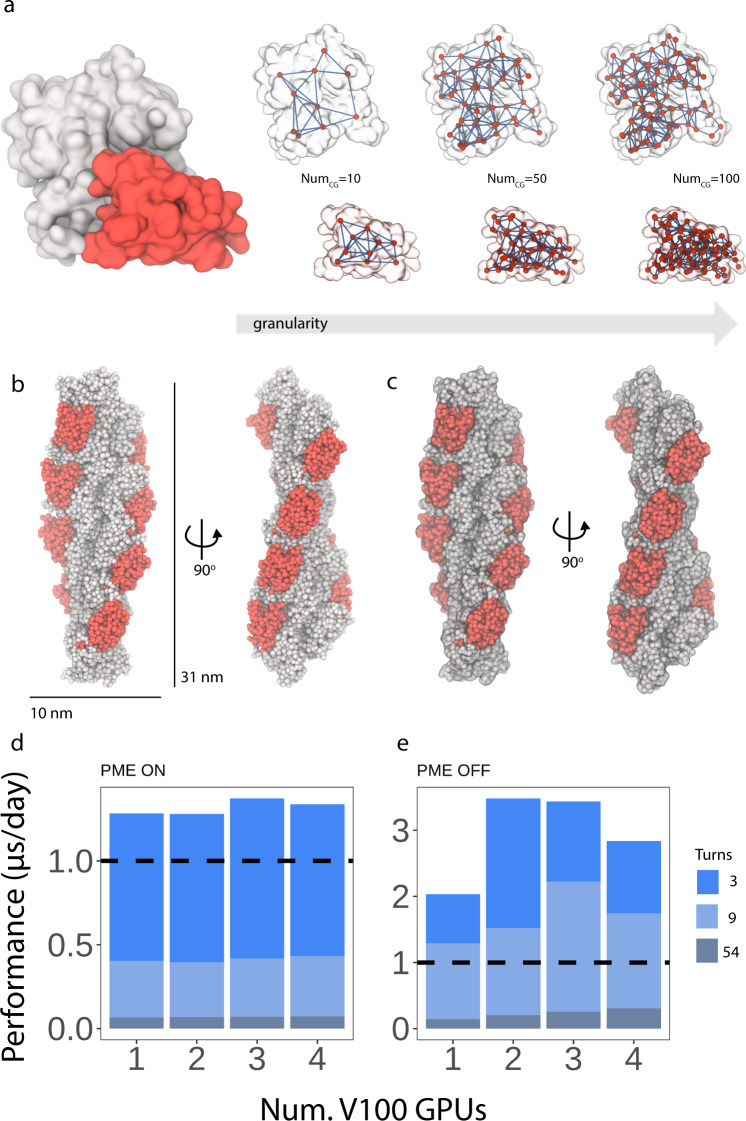
SBCG2 heteromultimeric cofilin-2 on actin filaments. **a** Left, atomistic surface representation of globular actin (white) bound to cofilin-2 (red). Right, corresponding SBCG2 representations of actin and cofilin-2, are shown superimposed with the atomistic molecular surfaces. **b** A single turn of the SBCG2 cofilin-2-bound actin filament, shown from two perspectives. One turn corresponds to a length of 31 nm. **c** The SBCG2 cofilin-2-bound actin filament is superimposed with the atomistic molecular surface, shown transparently. The shape of the atomistic filament is perfectly represented by the coarse model. **d** NAMD3 GPU benchmarks, utilizing NVIDIA V100s with PME on. **e** NAMD3 GPU benchmarks, utilizing NVIDIA V100s, with PME off. For panels **d** and **e**, we performed benchmark simulations with 3-, 9-, and 54-turn filaments to assess load-balancing and scaling with respect to system size. A legend is provided on the right. Benchmarks reported are the mean value of the six benchmark metrics reported by NAMD3^45^ for each simulation.

As mentioned in the introduction, a major goal of our coarse-graining endeavor is establishing a methodology that is transferable. Therefore, to determine the transferability of the SBCG2 methodology, we sought two unique molecular applications with the goal of simulating both homo- and heteromultimeric assemblies: HIV-1 CA, assembled into a conical capsid (Fig. 1) and cofilin-2 bound to actin filaments (Fig. 2). Direct application of the legacy SBCG implementation to these systems is hindered by hard limits in the number of beads, the lack of a metric to assess the inaccuracy of the CG mapping, and the lack of knowledge on how to determine accurate and optimal simulation parameters in modern architectures. The aforementioned SBCG2 systems were parameterized and subsequently subjected to finite-temperature molecular dynamics simulations using the fully GPU-resident NAMD3 molecular dynamics engine^45^ as described below.

Our first test system consisted of the HIV-1 capsid, a system we have characterized at atomistic resolution^46^ (EMDB 13422 and 13423). A full-scale conical capsid bound to the assembly co-factor inositol hexakisphosphate^46,47^ (Fig. 1b, c and Supplementary Fig. 5) was built from individual SBCG2-based HIV-1 CA monomers (Fig. 1a). The complete SBCG2 model of the conical capsid is described by 340,000 beads, representing a larger than 200-fold reduction in particles compared to the atomistic conical capsid of the same morphology (~77,000,000 atoms^46^). Molecular dynamics simulations of the SBCG2 model achieved greater than 1 µs per day sampling performance without particle mesh Ewald (PME)-based electrostatic evaluation^48^, and with PME enabled, the simulations sustained nearly 300 ns per day (Fig. 1d, e) performance. The latter performance represents a nearly threefold increase in peak performance over a CPU-only simulation employing ten compute nodes (Supplementary Fig. 2). For atomistic HIV-1 capsids, the capsid protein assembly equilibrates and reaches a stable configuration on the order of hundreds of nanoseconds^49^. Considering the latter, and with the 300 nanoseconds per day for the SBCG2 HIV-1 capsid, the time scales required for equilibration can be achieved in a matter of days on commodity hardware. Additionally, the performance of the SBCG2 conical capsid system scales across multiple NVIDIA V100/A100 GPUs and greatly broadens temporal resolution compared with molecular sampling on parallel CPU clusters (Fig. 1d, e, Supplementary Fig. 2, and Supplementary Table 1).

The second model used in the present work consisted of cofilin-2 bound to actin filaments^44^ (PDB 7U8K). This system represents a significantly different protein assembly compared to the conical capsid, at the physical and biochemical levels but also at the computational level. Spatial decomposition is an important aspect of parallel molecular dynamics simulation, namely in the evaluation of nonbonded electrostatics, where the spatial domain is discretized over multiple processing entities. After employing our framework to actin and cofilin-2 (Fig. 2a), we built heteromultimeric cofilin-2-bound actin filaments (Fig. 2b, c) of varying length ranging from a single turn (31 nm, 7000 beads) to a filament 54 turns in length (1.6 μm, 400,000 beads). Despite the spatial challenge presented by this system, where the ratio of length to the cross-sectional area is extremely large, we consistently exceeded 1 µs per day simulation performance with our three-turn filament system with PME-based electrostatic evaluation (Fig. 2d), but with no scaling regardless of filament size. Forgoing PME, we not only approach 4 microseconds per day performance, but we once again observe scaling across multiple GPUs (Fig. 2e). Optimizing the domain decomposition for PME-based evaluation is a future target of this work.

**Fig. 3:**
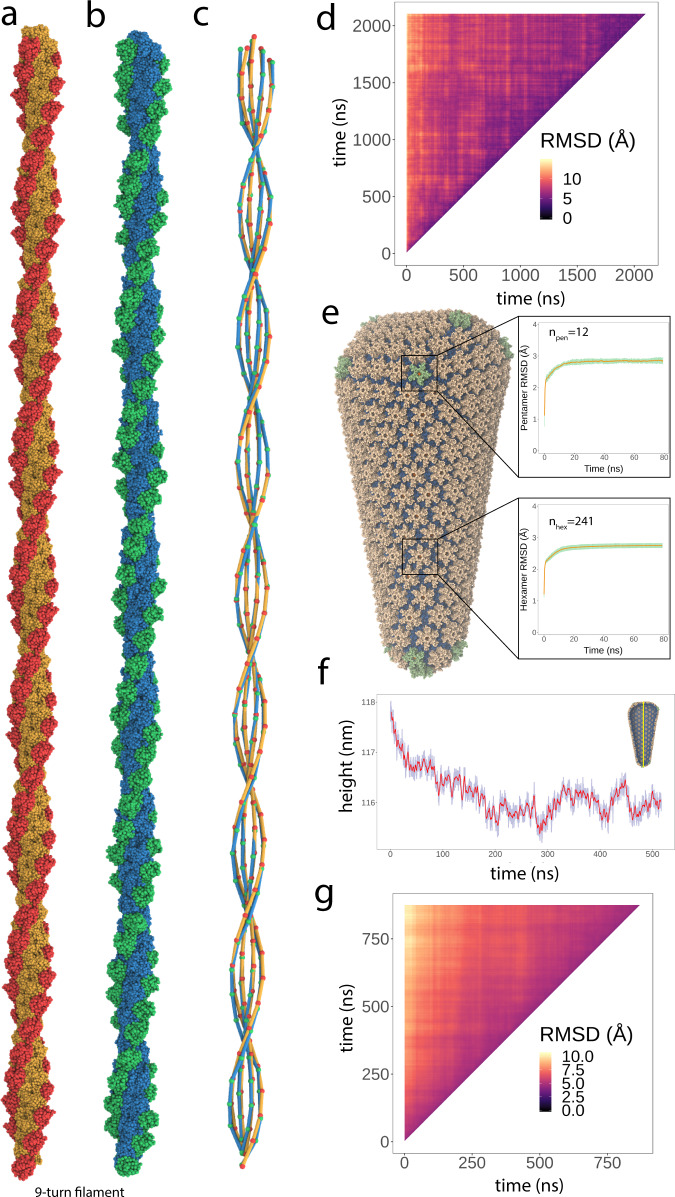
Stability of full-scale SBCG2 multimeric assemblies. **a**–**d** Cofilin-2 on actin filaments. **a** Initial state of the nine-turn cofilin-2-bound actin filament model, ~250 nm in length. **b** The nine-turn filament from panel a after unrestrained energy minimization, thermalization, equilibration, and ~200 ns of sampling at 298 K. **c** Visualization of the nine-turn filament’s helicity computed at two time points (panels **a** and **b**). For each cofilin-2 and actin subunit comprising the assembly, the center of mass is computed and a line is drawn to its sequential neighbor along the filament’s length. The inner and outer double helices represent actin and cofilin-2, respectively. Colors correspond to the states in panels **a** and **b**. The helical character of the filament is well-maintained throughout molecular sampling. **d** Pairwise RMSD heatmap of the entire three-turn filament (colored according to the legend provided). The analysis compares every pair of structures from a 2 µs SBCG2 trajectory, yielding a matrix where every element is the RMSD between two three-turn filaments. The filament achieves stability (<5 Å RMSD) after roughly 200 ns. **e**–**g** HIV-1 conical capsid. **e** Pentamer and hexamer RMSD analysis from the full-scale SBCG2 conical capsid. For an 80 ns equilibrium sampling trajectory, each capsomer was aligned to a single reference. The RMSD of each capsomer from the reference hexamer or pentamer was computed and the mean (orange) and standard deviation (green) was plotted across the trajectory. After approximately 20 ns, both hexamers and pentamers conform to the reference capsomer within 3 Å agreement. **f** Height time series of the conical capsid over 500 ns. The inset diagram illustrates the determination of height via the capsid’s principal axis of inertia. The capsid’s height converges after approximately 300 ns, and fluctuations <1 Å are seen thereafter. **g** Pairwise RMSD heatmap of the entire conical capsid (colored according to the legend provided). The analysis compares every pair of structures from a 900 ns SBCG2 trajectory, yielding a matrix where every element is the RMSD between two complete capsids. The analysis indicates that the capsid achieves stability (<5 Å RMSD) after roughly 300 ns.

Beyond establishing the performance of SBCG2 macromolecular assemblies, we analyzed our filamentous protein and conical capsid systems to establish their stability and, therefore, the efficacy of our approach. Importantly, for both systems, we specify no intermolecular, or otherwise empirical, interactions to maintain stability and assembly morphology. Figure 3a, b shows the nine-turn (280 nm, 69,000 beads) cofilin-2 bound actin filament at two time points. The left-handed helical character of filamentous actin is a defining morphological feature conferring biochemical significance^50^, which serves as a marker for SBCG2 assembly stability. The diagram shown in Fig. 3c represents a simplified view of the helicity of the filament at both the initial and final time points, colored accordingly, that enables visual inspection of helical character. We show that the filamentous quality, helical twist, and rise, are well-maintained without explicit steps to do so during model construction and optimization to enforce assembly morphology. Figure 3d shows a pairwise RMSD matrix for a 2 µs trajectory of the three-turn filament at 298 K. This analysis computes the RMSD between every possible pair of structures across the whole time series, and is well-suited for comparing large assembly constructs which undergo global fluctuations. Our analysis shows that the filament reaches global stability, fluctuating within 5 Å RMSD, after ~200 ns.

The HIV-1 conical capsid is an irregularly shaped, closed fullerenic shell. Similar to other retroviral capsids, the stability of the mature capsid manifests from intermolecular interfaces that are characterized by hydrophobicity and complementary charges. Without any empirical interactions to maintain stability, our SBCG2 capsids show marked structural stability. Locally, we quantify the stability of capsomers (hexamers and pentamers) via RMSD against a single reference capsomer, hexamer, or pentamer, and plot the mean and standard deviation of RMSD values versus time (Fig. 3e). Over ~20 ns of completely unrestrained sampling at 298 K, we observe capsomers achieving structural stability and converging to the reference capsomer (hexamer or pentamer) within 3 Å agreement. We employed several analyses of global behavior and stability. Figure 3f shows a trace of the capsid height over 500 ns of trajectory, computed by taking the minimum and maximum coordinates along the capsid’s principal axis of inertia. We see a small reduction in height over approximately 300 ns, then convergence, and fluctuations thereafter of <1 Å. The latter is consistent with what has been reported for a full-scale, atomistic capsid^49^. Utilizing 900 ns of sampling at 298 K, we compared every pair of frames to construct a pairwise RMSD matrix (Fig. 3g). This analysis computes the RMSD between entire SBCG2 capsid structures, with no omissions, showing that after ~300 ns, the capsid achieves consistent structural agreement <5 Å.

**Fig. 4:**
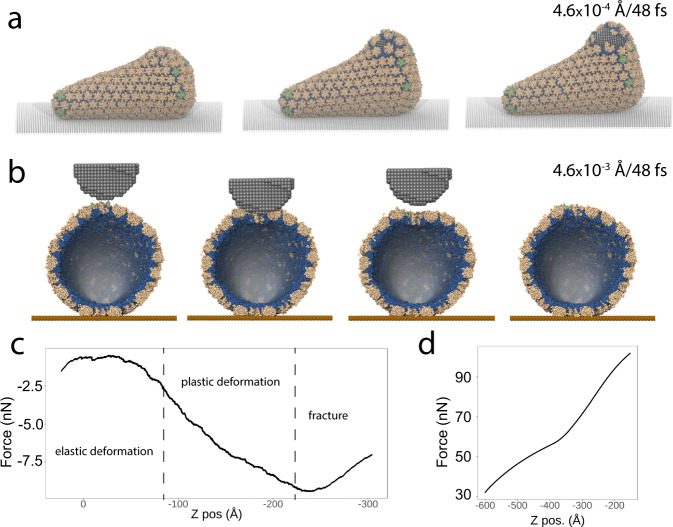
Application of shape-based coarse-graining 2 (SBCG2) to mechanical stress simulations of the HIV-1 conical capsid via constant velocity-steered molecular dynamics. **a** Snapshots of the capsid during internal rupture. First snapshot, where an internally-bound sphere of inert particles makes contact with the capsid surface and begins to deform the molecular surface. This deformation resides within the elastic deformation regime. The next snapshot in the sequence shows the beginnings of mechanical failure, once the capsid has deformed to an extent where fractures begin to manifest. The final snapshot shows the mechanical failure fully manifest, as the internally-bound probe punches through the capsid surface. **b** Snapshots of the capsid during nanoindentation. The initial state of the capsid, immediately prior to probe contact. The next snapshot shows the point of maximum deformation. Successively, retraction of the probe begins. In the final snapshot, the fully-recovered capsid is shown and the probe is out of view. **c** Force vs. Z (displacement) profile collected during internal rupture. This curve shows the evolution of forces through several viscoelastic regimes. **d** Force vs. Z (displacement) profile collected during nanoindentation, which utilized a tenfold increase in probe velocity. This curve is much smoother and displays a higher magnitude of forces acting on the probe, demonstrating the effect of velocity when performing such simulations. It should be noted that for both proof-of-concept simulations, the employed velocities, and therefore the measured forces, are significantly higher than what would be resolved with experimental AFM.

The adaptability and utility of SBCG2 modeling enables applications to mechanical stress and failure simulations of molecular containers via simulated atomic force microscopy (AFM). Previously, nanoindentation of low-granularity HBV capsids, constructed using the legacy SBCG method, was performed via constant velocity-steered molecular dynamics (SMD)^40,42^. More recently, utilizing Go models^51^, nanoindentation simulations were employed to determine the molecular details regarding the stability of the Norwalk virus capsid^10^. As a proof-of-concept for applications of our SBCG2 methodology that enables high-granularity modeling, we prepared simulations of our high-granularity HIV-1 conical capsid and subjected it to both nanoindentation and internal rupture. Using a spherical probe comprised of inert beads, we pulled the latter at a constant velocity, employing a rate of 0.00046 Å/48 fs time step for internal rupture (Fig. 4a), and a tenfold faster rate of 0.0046 Å/48 fs time step for nanoindentation (Fig. 4b). Remarkably, our internal rupture simulations and the measured force vs. displacement curve (Fig. 4c) show the evolution of forces across several viscoelastic deformation regimes. Snapshots in Fig. 4a show successive states, where the deformation transits through elastic, plastic, and mechanical failure regimes. With the latter revealing complete failure as the spherical probe punches through the capsid surface. For the nanoindentation simulation (Fig. 4b), we were interested in observing recoverable deformation of the capsid surface. Using a faster velocity, we pulled the probe to impose a shallow indent on the capsid surface, then retracted the probe away from the surface. Interestingly, the capsid fully recovers its original shape over a relatively short interval of 20 ns (Movie M4). While the probe velocity in the latter simulation is especially high, leading to forces in excess of 50 nano-Newtons (Fig. 4d), these simulations act as a proof-of-concept to what is possible using our SBCG2 methodology. As our understanding of the role of virus capsids continues to grow, as well as the forces and physical stresses imposed on capsids throughout the infection cycle, these sorts of structural perturbation simulations will provide valuable information on their mechanical behavior.

The high-granularity SBCG2 approach utilizes an unsupervised learning technique based on a topology representing network (TRN)^39^. By introducing exclusivity conditions during the initialization of neurons, we enable highly granular molecular modeling that was unachievable with the legacy implementation; therefore, requiring further steps to select and validate the model granularity. In addition, high-granularity models require the removal of overlapping degrees of freedom and parameterization of SBCG2 structures to match all-atom behavior. Altogether, SBCG2 suitably models large-scale macromolecules with remarkable simulation performance and greatly improves upon the legacy SBCG method, enabling new science. We present these developments in the subsections below. We elaborate on the complete SBCG2 modeling process, from molecular construction based on the TRN, including exclusivity conditions, to configuring high-performance simulations, in the Methods section.

### Granularity selection via charge density Fourier shell correlation

We aimed to utilize a quantitative metric to motivate and establish a basis for selecting model granularity. To this end, we employ Fourier shell correlation (FSC)^52^ between two charge density grids, one derived from the atomistic reference structure and the other resulting from SBCG2 mapping.

#### Computing charge densities

Charge densities are computed according to the charges on the molecular models, both atomistic and SBCG2. For the present study, we employ the VolMap plugin in VMD^53^. First, the structures are cast to a 3D voxel grid, with a grid spacing of 0.5 Å. Each atom in the structure is modeled as a normalized Gaussian distribution, with distribution widths equal to the van der Waals radii of the atoms or beads. The Gaussians in the grid are then additively distributed. The resultant grids store charge density in 3D space, which are amenable to FSC analysis. We employ the latter to gauge the fitness of our SBCG2 models to the atomistic reference from which it was derived.

#### Fourier shell correlation

Fourier shell correlation (FSC) is a commonly-employed method of measuring model-to-map fitness, map-to-map fitness, and other correlation quantities in electron microscopy modeling^52,54,55^. The charge density grid represents a discretized real-space array *f*(**n**) where the domain **n** = (*n*_1_, *n*_2_, *n*_3_) corresponds to the Cartesian axes, and where each voxel in the grid stores a charge value.

In order to measure the correlation between two charge density grids, their structure factors *F*(*r*) are first computed from the three-dimensional discrete Fourier transform (DFT)^56,57^. For the spatial domain **n** = (*n*_1_, *n*_2_, *n*_3_) of extent **N** = (*N*_1_, *N*_2_, *N*_3_), the DFT convolves *f*(**n**) into the reciprocal spatial frequency domain *r* (Å^−1^) as

1 $$F(r)=\mathop{\sum }\limits_{{{{{{{{\bf{n}}}}}}}}=0}^{{{{{{{{\bf{N}}}}}}}}-1}f({{{{{{{\bf{n}}}}}}}}){e}^{-2\pi ir\left(\frac{{{{{{{{\bf{n}}}}}}}}}{{{{{{{{\bf{N}}}}}}}}}\right)},$$

where $\frac{{{{{{{{\bf{n}}}}}}}}}{{{{{{{{\bf{N}}}}}}}}}=\left(\frac{{n}_{1}}{{N}_{1}},\frac{{n}_{2}}{{N}_{2}},\frac{{n}_{3}}{{N}_{3}}\right)$ and where $\mathop{\sum }\nolimits_{n=0}^{N-1}$ is the nested summation $\mathop{\sum }\nolimits_{{n}_{1}=0}^{{N}_{1}-1}\mathop{\sum }\nolimits_{{n}_{2}=0}^{{N}_{2}-1}\mathop{\sum }\nolimits_{{n}_{3}=0}^{{N}_{3}-1}$.

Following convolution, the two charge density grids, denoted as *F*_1_(*r*) and *F*_2_(*r*), are subjected to FSC analysis. FSC measures a normalized cross-correlation histogram, denoted here as *ζ*, across bins of increasing spatial frequency (visualized in Supplementary Fig. 9) as

2 $$\zeta (r)=\frac{{\sum }_{{r}_{i}\in r}{F}_{1}({r}_{i})\cdot {F}_{2}^{*}({r}_{i})}{\sqrt{{\sum }_{{r}_{i}\in r}|{F}_{1}({r}_{i}){|}^{2}\cdot {\sum }_{{r}_{i}\in r}|{F}_{2}({r}_{i}){|}^{2}}},$$

where ${F}_{2}^{*}({r}_{i})$ is the complex conjugate of *F*_2_(*r*_*i*_). Following the calculation of *ζ*, we evaluate the histogram at specific correlation values as *ζ*_*n*_, where *n* is a real number ∈ [0, 1], to derive a model resolution. A value of *n* = 0 indicates that the structure factors are entirely uncorrelated, whereas *n* = 1 indicates a perfect correlation between structure factors. Typically, the latter values of *n* are the so-called gold (0.143) and half (0.500) metrics. The assertion of resolution based on FSC will be elaborated in the following section.

Calculation of FSC in our SBCG2 methodology required the implementation of a GPU-accelerated FSC code within VMD, written in C++ and NVIDIA’s cuFFT library^57^. The new FSC implementation is native to VMD and is separate from the CGBuilder plugin. This routine is invoked from VMD’s Tcl interpreter with the command *measure fsc*. Our approach begins with an optional resampling step, then two out-of-place forward transforms of the input charge density grids, which are performed on available GPU hardware (eq. (1)). Next, correlation within each spatial frequency bin *r*_*i*_ is computed (eq. (2)). Returned is a two-column array, containing spatial frequency vs. FSC. The number of radial bins *N*_bins_ is determined as

3 $${N}_{{{{{{{{\rm{bins}}}}}}}}}=\frac{{N}_{1}}{2w},$$

where *N*_1_ is the largest and slowest-changing spatial extent, strided in memory, and where *w* is the Fourier shell width. The latter is set to the physical width of a single voxel in the input map, in units Å.

FSC calculation requires the spatial extent, voxel counts along each dimension, of the all-atom reference density and the coarse-grained charge density maps to be equal. Therefore, if the extent of the coarse-grained charge density map is different than the extent of the all-atom density map, the coarse-grain density map is resampled to fit the dimensions of the reference density.

For a given volumetric map of voxel counts *N*_1_, *N*_2_, *N*_3_, each voxel is identified by its three-dimensional indices, $\overrightarrow{v}=({v}_{1},\, {v}_{2},\,{v}_{3})$ with 0 ≤ *v* ≤ *N*_*i*_ ∀ *i* ∈ 1, 2, 3. The voxel coordinate $\overrightarrow{v}$ is related to the real-space Cartesian $\overrightarrow{x}=(x,\, y,\, z)$ coordinate representations by the 4 × 4 transformation matrix **M** as

4 $$\overrightarrow{x}=(x,\, y,\, z,\, 1)={{{{{{{\bf{M}}}}}}}}\cdot \overrightarrow{v}=\left(\begin{array}{cccc}\frac{{L}_{x}}{{N}_{1}-1}&0&0&{C}_{x}\\ 0&\frac{{L}_{y}}{{N}_{2}-1}&0&{C}_{y}\\ 0&0&\frac{{L}_{z}}{{N}_{3}-1}&{C}_{z}\\ 0&0&0&1\end{array}\right)\cdot \left(\begin{array}{c}{v}_{1}\\ {v}_{2}\\ {v}_{3}\\ 1\end{array}\right),$$

where *L*_*x*_, *L*_*y*_, *L*_*z*_ are the physical extents of the map, in units Å, and *C*_*x*_, *C*_*y*_, *C*_*z*_ is the origin, also in units Å. Consequently, the inverse transform can be applied to yield a voxel coordinate as $\overrightarrow{v}={{{{{{{{\bf{M}}}}}}}}}^{-1}\cdot \overrightarrow{x}$. Note that the voxel width, *w*, of the map is encoded in the *L* terms, since *L*_*d*_ = *w* ⋅ *N*_*d*_.

For our resampling procedure, each voxel coordinate ${\overrightarrow{v}}_{{{{{{{{\rm{aa}}}}}}}}}=({v}_{{{{{{{{\rm{aa}}}}}}}},1},\, {v}_{{{{{{{{\rm{aa}}}}}}}},2},\, {v}_{{{{{{{{\rm{aa}}}}}}}},3})$ in the all-atom charge density map is transformed into its real-space (Å) Cartesian representation $\overrightarrow{x}=(x,\, y,\, z)$ via the transformation matrix **M**_*aa*_ of the all-atom map: $\overrightarrow{x}={{{{{{{{\bf{M}}}}}}}}}_{{{{{{{{\rm{aa}}}}}}}}}\cdot {\overrightarrow{v}}_{{{{{{{{\rm{aa}}}}}}}}}$. Then, the voxel element in the coarse-grained map that contains this Cartesian coordinate is calculated using the inverse transformation matrix ${{{{{{{{\bf{M}}}}}}}}}_{cg}^{-1}$ for the coarse-grained map: ${\overrightarrow{v}}_{{{{{{{{\rm{cg}}}}}}}}}={{{{{{{{\bf{M}}}}}}}}}_{{{{{{{{\rm{cg}}}}}}}}}^{-1}\cdot \overrightarrow{x}$. This voxel element in the coarse-grained map ${\overrightarrow{v}}_{{{{{{{{\rm{cg}}}}}}}}}$ is then used as the center for trilinear interpolation. In cases where the computed Cartesian coordinate does not map to a voxel index in the coarse-grained map, a charge value of zero is assigned. The resulting interpolated charges are then stored in the resampled map with the same spatial extent as the all-atom map, as required for FSC calculation. Note that **M**_aa_ and **M**_cg_ are defined equivalently (eq. (4)) but have different spatial extents and centers prior to resampling; the subscripts reflect this point.

To validate our FSC implementation, we performed a set of FSC analyses using our implementation *measure fsc* and the widely-used EMAN2 software^56^ (Supplementary Fig. 10). All tests utilized the same input densities, as well as a pre-processing resampling step as discussed above. For the EMAN2 test cases, we utilized UCSF Chimera^58^ for resampling, whereas the *measure fsc* test cases utilized our built-in, VMD-native resampling procedure described above (eq. (4)). The results show that our GPU-accelerated *measure fsc* method yields identical results to EMAN2, with root-mean-square error on the order of 10^−7^ and 10^−8^ (Supplementary Fig. 10), well within floating point error tolerance. Our C++ implementation is high-performance and conveniently invoked directly within a VMD session, requiring no additional software or libraries.

#### Model selection based on charge density correlation

**Fig. 5:**
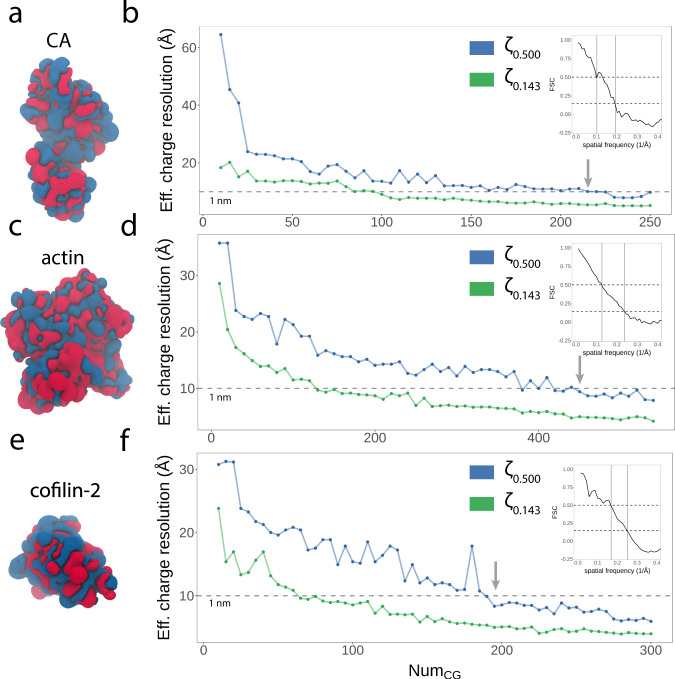
FSC analysis of SBCG2 granularity vs. effective charge density resolution. **a** Charge density of the all-atom reference structure of HIV-1 CA. Regions of positive and negative charge density are colored blue and red, respectively. **b** Effective charge density resolutions via FSC for models Num_CG_ ∈ [10, 250], plotted with two metrics: *ζ*_0.143_ and *ζ*_0.500_, green and blue, respectively. The dotted gray line represents a resolution of 1 nm, and the gray arrow indicates the first sub-nanometer model in the series. The inset plot shows the FSC vs. spatial frequency trace for HIV-1 CA with Num_CG_ = 250. **c** Charge density of the all-atom reference structure of actin and **d** corresponding effective charge density analysis for actin models with Num_CG_ ∈ [10, 540]. The inset plot corresponds to the FSC vs. spatial frequency trace for actin with Num_CG_ = 540. **e** Charge density of the all-atom reference structure of cofilin-2 and **f** corresponding effective charge density analysis for cofilin-2 models with Num_CG_ ∈ [10, 300]. The inset plot corresponds to the FSC vs. spatial frequency trace for cofilin-2 with Num_CG_ = 540.

To assess the benefits of increased granularity with respect to accurately representing an atomistic charge density, we utilized our HIV-1 CA (Fig. 5a, b), actin (Fig. 5c, d), and cofilin-2 (Fig. 5e, f) structures and computed sets of SBCG2 models ranging from low to high granularity. The atomistic reference and each of the SBCG2 models were subjected to charge density calculation as outlined above, with special care taken to ensure that the van der Waals radii of the SBCG2 models were properly asserted before casting charges to the 3D voxel grid (Fig. 2c), since charge density depends on vdW radius (see Computing charge densities).

To interpret the analysis, we employ the *ζ*_0.143_ and *ζ*_0.500_ metrics (Fig. 5b, d, f), commonly used to estimate the resolution of particle reconstructions from electron microscopy. Metrics to determine reconstruction resolution are a subject of significant study and debate^59,60^. In general, the FSC analysis considers amplitudes in structure factors at increasing radii of spatial frequency or inverse resolution (Å^−1^)^55,61^. The point along the spatial frequency axis at which the correlation of two structure factors diminishes steeply is used for resolution determination^62^. The two metrics, *ζ*_0.143_ and *ζ*_0.500_, have each been argued as effective methods of determining resolution, and additional analyses, such as the ResLog plot, have been put forth to ensure accurate particle alignment, free of aberrant correlation^60^. In our case, we are assessing the correlation among two charge density grids, where charges are interpolated from structures with differing granularity.

Our motivation for utilizing FSC is to assert an optimal granularity for representing the reference charge density with <10 Å correlation, while adding as few degrees of freedom as possible and thus limiting the computational expense of subsequent simulations. Trivially, a CG model with one representative bead per atom, and thus a one-to-one mapping of charge to each bead, would be perfectly correlated. Our results indicate that SBCG2 models for HIV-1 CA fall below 10 Å resolution in excess of 210 beads, employing the more stringent *ζ*_0.500_ metric. For actin, the first sub-nanometer model in the series was found to consist of 450 beads, and for cofilin-2, 195 beads. Supplementary Figs. 11, 12, 13 show additional details of this analysis for CA, actin, and cofilin-2, respectively, with examples of SBCG charge densities and additional FSC vs. spatial frequency traces.

Based on our analysis and subsequent determination of a correlation of <10 Å, we created a model of HIV-1 CA containing 221 beads, representing one bead per protein residue for the CA sequence utilized. For actin and cofilin, we chose 500 and 270-bead models, respectively, representing an approximately equal ratio of atoms to beads for each structure. While we construct and optimize SBCG2 models separately, the latter choice was made in anticipation of adjoining the models to constitute the heteromultimeric assembly. We then proceeded to the critical step of parameterizing the bond and angle terms governing the model, which are essential for accurately reproducing dynamics.

### Parameterizing sub-nanometer SBCG2 models

Parameterization of the SBCG2 bond and angle terms is accomplished with Boltzmann inversion, which is a technique commonly utilized^40–43,63–65^. Boltzmann inversion is employed to derive force constants based on mean square displacement (MSD, eq. (6)) of bonds and angles during all-atom simulation. For parameterizing HIV-1 CA, we employed an all-atom simulation of an HIV-1 CA trimer of dimers (Supplementary Fig. 6a), the latter constructed from six CA monomers. While we parameterize only a single SBCG2 CA monomer, the benefit of utilizing an assembly construct for inversion is threefold. First, the aggregate sampling of the atomistic trajectory totals nearly half a microsecond, 480 ns; second, the corresponding SBCG2 trimer of dimers (Supplementary Fig. 6b), simulated throughout iterative refinement, provides ample opportunity for cross-validation throughout the process; and third, to preserve the dynamical behavior of the CA monomers (Supplementary Fig. 6c) in their assembly environment given the state-dependence of Boltzmann inversion. For actin and colifin-2, we utilized a similar approach, performing an all-atom simulation of a single globular actin bound to one human cofilin-2 protein^44^.

**Fig. 6:**
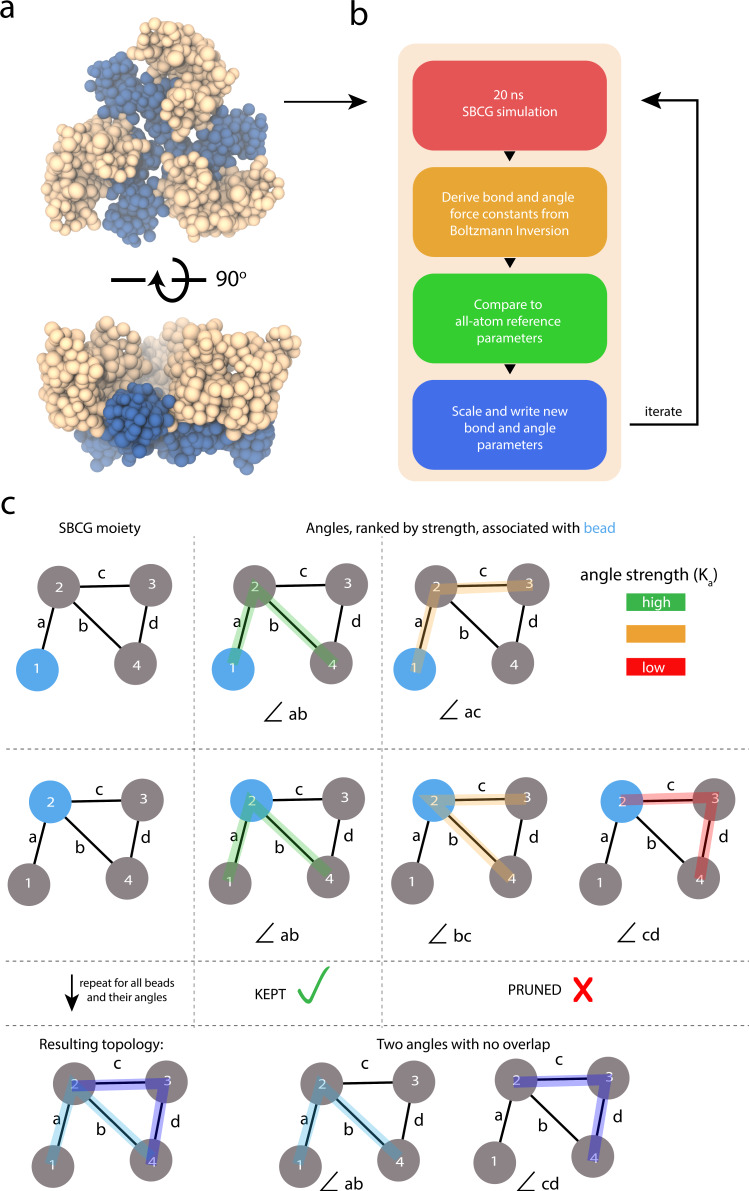
Graphical overview of our SBCG2 model refinement protocol. **a** SBCG2 HIV-1 CA trimer of dimers, utilized for successive 20 ns simulations during iterative refinement. The N-terminal domain is colored tan, and the C-terminal domain is colored blue. **b** The iterative parameter refinement procedure via Boltzmann inversion. For one iteration, 20 ns of equilibrium sampling is collected at 298 K. Next, bond and angle force constants are derived via Boltzmann inversion (eq. (5)). Parameters derived from the SBCG2 model simulation are then compared to the all-atom reference parameters and are scaled by their error (eq. (7)). Finally, new bond and angle parameters are written and employed for the succeeding refinement iteration. **c** Graphical example of the pruning procedure employed in refining our model. This example shows an SBCG2 structure of four beads enumerated 1 through 4 and four bonds a through d. Initially, angles are determined exhaustively based on the bonded connectivity. For each bead, we rank its associated angle parameters by their constants, *K*_*a*_, and keep only the strongest parameter. This example demonstrates that our algorithm permits two beads to share the same force constant, if it is deemed the strongest for each bead.

In the following subsections, the formulation of Boltzmann inversion, the iterative refinement protocol (Fig. 6a, b), and the necessary considerations for optimization of sub-nanometer structures, particularly the removal of overlapping degrees of freedom (Fig. 6c), are discussed.

#### Boltzmann inversion from atomistic simulation

Boltzmann inversion derives force constants for bonds and angles, *K*_*b*_ and *K*_*a*_, respectively, according to

5 $${K}_{b,a}=\frac{{k}_{B}T}{2{D}_{b,a}}$$

where

6 $${D}_{b,a}=\left\langle {r}_{b,a}^{2}\right\rangle -{\langle {r}_{b,a}\rangle }^{2},$$

and where *r*_*b*_ and *r*_*a*_ are the measured bond and angle values. Units of *K*_*b*_ and *K*_*a*_ are kcal *mol* ⋅ Å^−2^ and kcal mol ⋅ rad. ^−2^, respectively. *k*_B_ is the Boltzmann constant and *T* is the absolute temperature, in units of Kelvin.

**Fig. 7:**
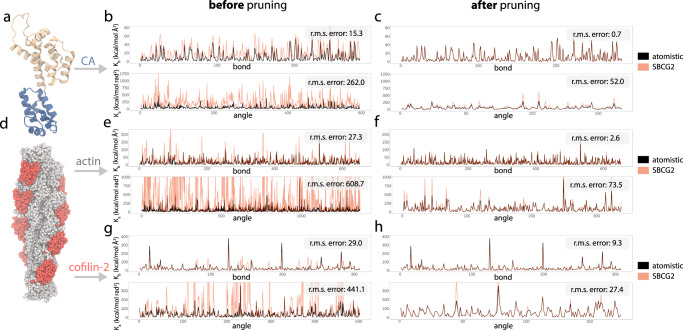
SBCG2 bond and angle parameter optimization results for HIV-1 CA, actin, and cofilin-2. **a** Monomeric HIV-1 CA all-atom structure, shown in cartoon representation. **b**, **c** Corresponding bond and angle parameter fits following from iterative Boltzmann inversion. The black traces show the atomistic bond and angle parameter trace as computed via Boltzmann inversion from the all-atom reference trajectory, and orange the SBCG2 bond and angle parameter trace via Boltzmann inversion from the simulation corresponding to the final refinement iteration **b** before pruning and **c** after pruning. **d** Atomistic surface representation of cofilin-2 bound to one turn of actin. **e**, **f** Actin bond and angle parameter fits following from iterative Boltzmann inversion **e** before pruning and **f** after pruning. **g**, **h** Cofilin-2 bond and angle parameter fits following from iterative Boltzmann inversion **g** before pruning and **h** after pruning. The root-mean-square error between the SBCG2 bond and angle parameters and their respective all-atom reference parameters are annotated within each plot.

After the initial derivation of bond and angle force constants from the all-atom simulation, we performed an SBCG2 simulation with the resulting parameters, and utilized Boltzmann inversion targeting the SBCG2 trajectory as validation; we observed a terrible fit (Fig. 7 and Supplementary Fig. 14a). This behavior of the Boltzmann inversion method has been reported elsewhere^42,64^ and is a known short-coming of this approach. The problem is in the assumption that each bond and angle are independent. In reality, bonds and angles are highly coupled throughout the structure and this is especially true in the sub-nanometer SBCG2 regime. To remedy this, we employ an iterative refinement protocol, based on the previous work^64^.

#### Iterative refinement

From refinement iteration *i*, the parameters for the next iteration *i* + 1 are computed according to

7 $${K}_{b,i+1}={K}_{b,i}-m\left({K}_{b,aa}-{K}_{b,i}\right),\quad {{{{{{{\rm{and}}}}}}}}\,{K}_{a,i+1}={K}_{a,i}-n\left({K}_{a,aa}-{K}_{a,i}\right).$$

*K*_*b*,*aa*_ and *K*_*a*,*aa*_ are the bond and angle force constants derived from the all-atom reference trajectory. The constants *K*_*b*,*i*_ and *K*_*a*,*i*_ are derived from Boltzmann inversion of 20 ns SBCG2 simulations. Variables *m* and *n* are scaling constants and are treated as hyperparameters^64^.

Prior to deploying the above protocol, we performed a parameter sweep to identify optimal *m* and *n* scaling parameters. The sweep covered *m* and *n* ∈ [0.1, 0.9] with a stride of 0.1 for each constant, resulting in 81 separate SBCG2 simulations 20 ns in length. Inversion was then applied to these trajectories to yield parameters, and the improvement from the previous parameter set was measured via root-mean-square error (RMSE) (Supplementary Fig. 7).

With optimal *m* and *n* scaling constants identified, we performed many iterations of refinement. After, it became clear that the parameters had improved, but converged to an unphysical state with the poor fit (Movie M2). For all three of our structures, angle parameters were particularly problematic. We determined that the problem is caused by high connectivity, and, therefore redundant degrees of freedom (Fig. 7b, e, g and Supplementary Fig. 14b, c).

#### Pruning redundant degrees of freedom

Supplementary Fig. 14 shows the analysis utilized to identify the cause of unphysical convergence. For each angle parameter, comprised of three CG beads, we analyzed the connectivity associated with the beads. Angle parameters with the poorest fit were found to involve beads with high connectivity. Conversely, we found that angle parameters involving CG beads with relatively few bonded terms were well-fit. Regions exemplary of the latter are shaded with red and green, respectively, in Supplementary Fig. 14c.

Further, we analyzed violations, i.e., deviations of the SBCG2 angle vs. its all-atom reference value, and found that the behavior of a given CG bead, and therefore its bond and angle parameters, is dominated by its strongest connections; weak parameters are overpowered by stronger, coupled parameters, and thus a violation is manifest. The latter, coupled with regions of the topology containing many overlapping, redundant degrees of freedom, led to an untenable optimization problem. To remedy this, we collected for each CG bead the angle parameters with which it is associated. For each bead, only the strongest angle parameter, i.e., the parameter with the highest force constant based on all-atom reference simulations, was retained. We refer to this process as pruning (Fig. 6c).

#### Converged fit

Following the pruning of redundant angle parameters, our optimization immediately converged to a better fit for all three of our structures (Fig. 7c, f, h). While not scale-invariant, we employ root-mean-square error (RMSE) as a progress indicator of the fitting. The plots in Fig. 7 are annotated with the bond and angle RMSE for each of our three structures, before and after pruning, quantifying how crucial removing redundant degrees of freedom is. Prior to any sub-nanometer SBCG2 parameter optimization endeavor, pruning should be performed because the optimization, based on the present formulation where bonds and angles are treated independently, is otherwise untenable in high-granularity cases, as we have demonstrated with three unique structures. Our pruning algorithm (Fig. 6) is available for easy use within the CGBuilder plugin, distributed with VMD^53^.

Overall, our SBCG2 methodology constitutes a next-generation shaped-based coarse-graining procedure, for efficient simulation of large biomolecular assemblies and have outlined the protocol for its effective deployment. SBCG2 overcomes many of the limitations of its predecessor legacy SBCG^39–43^, and includes a FSC method for granularity selection and coarse-grained model force field parameter derivation, as illustrated in Supplementary Fig. 1. In particular, SBCG2 enables high granularity coarse-grained modeling. To facilitate the latter, we developed a VMD-native GPU-accelerated FSC method. Optimization of parameters, as well as the removal of redundant degrees of freedom, are outlined and illustrated in detail to reproduce atomistic behavior. We describe numerous considerations for configuring and performing simulations of biomolecular assemblies using sub-nanometer SBCG2, such as temperature control, computation of the integration time step, and long-range electrostatics. Our code is freely-available as part of the CGBuilder plugin in VMD 1.9.4^53^, which is distributed with a corresponding tutorial and example files.

## Methods

Shape-based coarse-graining (SBCG) is a modality of CG modeling which maps the coordinates of CG beads according to the shape or topology of an atomistic input. Legacy SBCG has been successfully employed to study the stability and deformation of viral capsids^40–42^ as well as the mechanisms of lipid membrane remodeling by proteins^43,64^. For completion, we will elaborate on the theoretical underpinnings of the topology representing the neural network, developed elsewhere^39^ and employed in the legacy SBCG implementation for molecular topology learning. Then, we will elaborate on advances to the TRN that enable high-granularity SBCG2 modeling.

The conceptual back end of this method is a topology representing a neural network^39^. The topology representing the network employs a Hebbian adaptation rule with winner-take-all competition (eq. (11)) to determine algorithmically the positions of CG beads relative to an atomistic input. Formally, this procedure constructs a Voronoi tessellation in ${{\mathbb{R}}}^{3}$^39^, where each polyhedron in the tessellation represents a CG bead. The emerging Voronoi polyhedra partition atoms of the input structure, and their properties are applied to the CG bead positioned at the partition’s center of mass. The latter is detailed in the forthcoming sections.

### Machine-learning-based molecular topologies with competitive Hebbian adaptation

A detailed mathematical description of the topology representing network (TRN) is presented elsewhere^39^. Here, we will first introduce basic nomenclature, then the most relevant concepts in the context of molecular topology learning, including Hebbian adaptation, Delaunay Triangulations, and finally, the algorithmic formulation of the TRN itself.

For a set of neural units *i* = 1, …, *N*, lateral connections can form between any *i* to another, referred to as *j*. These lateral connections represent synaptic links, and are described by a matrix **C** containing connections ${C}_{ij}\in {{\mathbb{R}}}_{0}^{+}$. The larger an element *C*_*ij*_ is, the stronger the synaptic link between *i* and *j*. A connection is manifest only when *C*_*ij*_ > 0; if *C*_*ij*_ ≤ 0 then *i* and *j* are disconnected.

Hebb’s postulate states that a pre-synaptic unit *i* shares a synaptic link with post-synaptic unit *j* if the two neural units are concurrently active. Originally formulated as a governing description of the neurological architecture of the hippocampus^66^, Hebb’s rule can be represented as

8 $$\Delta {C}_{ij}\propto {y}_{i}\cdot {y}_{j}.$$

That is, the change in the strength of the link between neural units *i* and *j*, Δ*C*_*ij*_, is proportional to the pre- and post-synaptic activity of the *i* and *j* pair. The relation in equation (8) is augmented with weight vectors ${\{{{{{{{{\bf{w}}}}}}}}\}}_{i=1}^{N}$ such that every neural unit *i* has a corresponding weight vector ${{{{{{{{\bf{w}}}}}}}}}_{i}\in {{\mathbb{R}}}^{D}$ which describes the center of the receptive field for neuron *i*. In this setting, the receptive field is the same as in other learning applications: it describes a region of the input space that is sensory, or responsive to stimuli, and maps to a corresponding feature or activation in the output space^67^. For constant input patterns ${{{{{{{\bf{v}}}}}}}}\in {{\mathbb{R}}}^{D}$, the activation of neural unit *i*, *y*_*i*_, is larger the closer its **w**_*i*_ is to **v**^39^.

Introducing a positive, continuous, and monotonically decreasing function *R*(⋅) such that *y*_*i*_ = *R*(∣∣**v** − **w**_*i*_∣∣), which describes the receptive field, we rewrite equation (8) as^39^

9 $$\Delta {C}_{ij}\propto R(||{{{{{{{\bf{v}}}}}}}}-{{{{{{{{\bf{w}}}}}}}}}_{i}||)\cdot R(||{{{{{{{\bf{v}}}}}}}}-{{{{{{{{\bf{w}}}}}}}}}_{j}||).$$

Connection strengths *C*_*ij*_ are then solved by integrating equation (9) over the given pattern distribution *P*(**v**) as

10 $$\Delta {C}_{ij}(t\to \infty )\propto {\int}_{{{\mathbb{R}}}^{D}}R(||{{{{{{{\bf{v}}}}}}}}-{{{{{{{{\bf{w}}}}}}}}}_{i}||)\cdot R(||{{{{{{{\bf{v}}}}}}}}-{{{{{{{{\bf{w}}}}}}}}}_{j}||)d{{{{{{{\bf{v}}}}}}}}.$$

Evidently, equation (10) establishes a connection strength based simply on the area of overlap between the receptive fields of neural units *i* and *j*. Because the formulation of the receptive field *R*(⋅) is continuous and monotonically decreasing as ∣∣**v** − **w**_*i*_∣∣ increases, elements *C*_*ij*_ of **C** connect all neurons to one another. In ref. ^39^, the authors introduce the notion of winner-take-all selection to Hebb’s rule (eq. (8)).

The competitive Hebb’s rule with winner-take-all selection becomes

11 $$\Delta {C}_{ij}=\left\{\begin{array}{ll}{y}_{i}\cdot {y}_{j}\quad &{{{{{{{\rm{if}}}}}}}}\,{y}_{i}\cdot {y}_{j}\ge {y}_{k}\cdot {y}_{l}\,\forall k,\, l=1,\ldots,\, N\\ 0\quad &{{{{{{{\rm{otherwise}}}}}}}}.\end{array}\right.$$

Enforcing adaptation via equation (11) rather than equation (8) results in connectivity **C** that corresponds to the Delaunay triangulation of the weight vectors **w**. Importantly, the original authors proved that, for a sequentially presented distribution of input patterns *P*(**v**) with support everywhere on ${{\mathbb{R}}}^{D}$, elements *C*_*ij*_ of **C** obey *θ*[*C*_*ij*_(*t* → *∞*)] = *A*_*ij*_ in the asymptotic limit. *θ*(⋅) is the Heavyside step function and *A*_*ij*_ are elements of the adjacency matrix **A** of the Delaunay triangulation^39^. Here, the Delaunay triangulation is defined as the graph connecting weights **w**_*i*_ and **w**_*j*_ with adjacent Voronoi polyhedra *V*_*i*_ and *V*_*j*_.

#### Algorithm 1

(i) Initialize all connections *C*_*ij*_ to zero;

(ii) Present input pattern ${{{{{{{\bf{v}}}}}}}}\in {{\mathbb{R}}}^{D}$ with distribution *P*(**v**);

(iii) Find unit *i* for which

$$||{{{{{{{\bf{v}}}}}}}}-{{{{{{{{\bf{w}}}}}}}}}_{i}||\le||{{{{{{{\bf{v}}}}}}}}-{{{{{{{{\bf{w}}}}}}}}}_{k}||\,\forall k=1,\ldots,\, N$$

and unit *j* for which

$$||{{{{{{{\bf{v}}}}}}}}-{{{{{{{{\bf{w}}}}}}}}}_{j}||\le||{{{{{{{\bf{v}}}}}}}}-{{{{{{{{\bf{w}}}}}}}}}_{k}||\,\forall k \, \ne \, i,\, k=1,\ldots,\, N;$$

(iv) If *C*_*ij*_ = 0, set *C*_*ij*_ > 0 (connect *i* and *j*); else, leave *C*_*ij*_ unchanged. Repeat at (ii).

Theorem 1 in ref. ^39^ contains the associated proof that *A*_*ij*_ = *θ*(*C*_*ij*_) is equivalent to the adjacency matrix of the Delaunay triangulation ${{{{{{{{\mathcal{D}}}}}}}}}_{S}$ constructed from the set of weights $S={\{{{{{{{{\bf{w}}}}}}}}\}}_{i=1}^{N}$.

Finally, we will introduce the topology-preserving map, and particularly we will explain how competitive Hebbian adaptation, as outlined above, is employed for molecular topology modeling. So far, we have operated under the assumption that *P*(**v**) has support on the entire embedding space ${{\mathbb{R}}}^{D}$. For many real-world input patterns, such as molecular coordinates, the input *P*(**v**) does not have support everywhere, but rather only on a submanifold $M\subset {{\mathbb{R}}}^{D}$. Competitive Hebb’s rule (eq. (11)) forms a subgraph of the complete Delaunay triangulation in these instances, which remains topology preserving^39^.

**Fig. 8:**
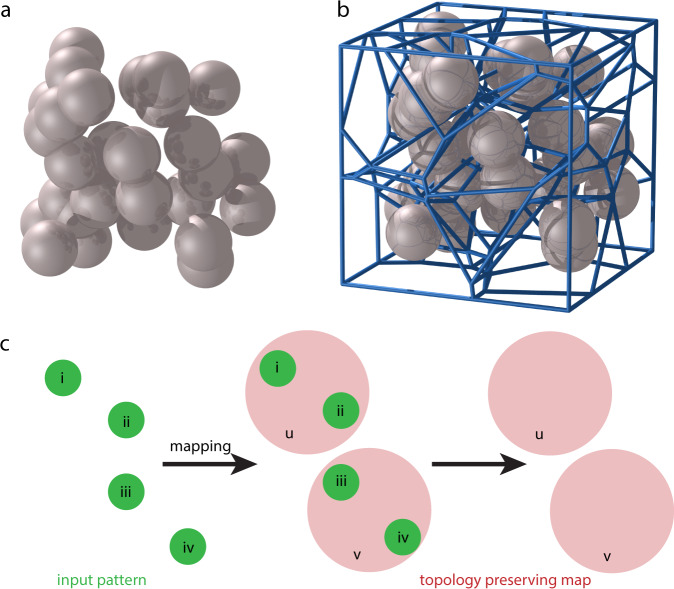
Topology-preserving maps via 3D Voronoi tessellation. **a** A 30-point cloud in 3D, generated randomly in a cubic domain. **b** Resulting 3D Voronoi tessellation of the point cloud in panel **a**. Each Voronoi cell partitions the spatial domain into regions that are closer to a given point than any other. Voronoi tessellation was performed with the *voro++* command-line tool^77^ and rendering was performed with the Persistence of Vision raytracer^78^. **c** Visual depiction and definition of a topology-preserving map. The input pattern (green) consists of four points, enumerated i–iv. The coarse mapping groups two input points into a single coarse point (red), named u and v. The resulting mapping is topology preserving if adjacent features in the input pattern are adjacent in the output map. In this case, coarse point u maps to input points i and ii; coarse point v maps to points iii and iv; u and v bound adjacent groups of the input pattern and are adjacent in the output map.

The topology-preserving map is described by a mapping Φ that projects features from a manifold *M* onto the neural units *i* = 1, …, *N* comprising a graph *G*. The mapping is directed by the set of weights ${\{{{{{{{{\bf{w}}}}}}}}\}}_{i=1}^{N}$ such that features of the input pattern **v** ∈ *M* are mapped to the most proximal neural unit, graph vertex, *i*. Recall that each unit *i* has an associated weight **w**_*i*_, describing its receptive field. The notation i^*^(**v**) clarifies that the resulting Voronoi polyhedron *V*_*i*_ associated with unit *i* of graph *G* completely bounds the feature **v**. The mapping is expressed as

12 $${\Phi }_{S}:M\to G,\quad {{{{{{{\bf{v}}}}}}}}\in M\to {i}^{*}({{{{{{{\bf{v}}}}}}}})\in G,$$

where the inequality

13 $$||{{{{{{{{\bf{w}}}}}}}}}_{{i}^{*}({{{{{{{\bf{v}}}}}}}})}-{{{{{{{\bf{v}}}}}}}}||\le||{{{{{{{{\bf{w}}}}}}}}}_{i}-{{{{{{{\bf{v}}}}}}}}||\,\forall i\in G$$

establishes the mapped vertex. The mapping Φ_*S*_ is topology preserving if adjacent features **v** ∈ *M* correspond to adjacent vertices of *G*, and therefore coincide with adjacent associated weights and resulting Voronoi polyhedra (Fig. 8c). To satisfy this requirement, algorithm 1 is amended to include an additional step to adjust weights ${\{{{{{{{{\bf{w}}}}}}}}\}}_{i=1}^{N}$ according to the neural gas algorithm^68^. The latter introduces an age *t*_*ij*_ for each connection, and is used to remove elements *C*_*ij*_ corresponding to weights and receptive fields that are no longer adjacent following evolution. The final formulation of the TRN algorithm is:

#### Algorithm 2

(i) Initialize each weight **w**_*i*_ for *i* = 1, …, *N*, and set all connections *C*_*ij*_ to zero;

(ii) Present a pattern **v** ∈ *M*, where each **v** is drawn with equal probability;

(iii) For each *i* determine the number *n*_*i*_ of units *j* where

$$||{{{{{{{\bf{v}}}}}}}}-{{{{{{{{\bf{w}}}}}}}}}_{j}||\le||{{{{{{{\bf{v}}}}}}}}-{{{{{{{{\bf{w}}}}}}}}}_{i}||;$$

(iv) Evolve **w**_*i*_ by the neural gas algorithm^68^

$${{{{{{{{\bf{w}}}}}}}}}_{i}^{new}={{{{{{{{\bf{w}}}}}}}}}_{i}^{old}+\epsilon \cdot {e}^{-{n}_{i}/\lambda }\left({{{{{{{\bf{v}}}}}}}}-{{{{{{{{\bf{w}}}}}}}}}_{i}^{old}\right)\,i=1,\ldots,\, N;$$

(v) If *C*_*ij*_ = 0, set *C*_*ij*_ > 0 (connect *i* and *j*) and set *t*_*ij*_ = 0; else, leave *C*_*ij*_ unchanged and set *t*_*ij*_ = 0;

(vi) Increment the age of all other connections made to unit *i*;

(vii) Remove connections made to unit *i* that exceed a predefined age threshold; repeat at (ii).

Hyperparameters *ϵ* and *λ* in step (iv) above are explained, as well as guidance for setting their values, in ref. ^39^.

In summary, the TRN computes a Delaunay triangulation from a set of weights that represent the locality of graph vertices and neural units relative to features in the input space. For SBCG model building, each desired CG bead is treated as a neural unit, and its initial weight is a Cartesian coordinate within the embedding domain. Input patterns, i.e., atomic coordinates, are drawn sequentially from the reference molecule in a step-wise fashion. At each step, the weight associated with each neural unit is adapted until it is closer to its respective input pattern (atomic domain within the reference molecule) than any other. Movie M1 demonstrates the complete adaptation process using HIV-1 CA.

#### Mapping of atomic properties to an SBCG model

Following optimization of the topology representing the network, the properties of *N*_cell_ atoms within each Voronoi cell are mapped to CG beads. For a CG bead *j*, its mass *M*_*CG*,*j*_ is computed according to

14 $${M}_{CG,j}=\mathop{\sum }\limits_{i=1}^{{N}_{{{{{{{{\rm{cell}}}}}}}}}}{m}_{aa,i},$$

where *m*_*aa*,*i*_ is the mass of atom *i* in the given Voronoi cell *j*.

Assignment of charge *Q*_*CG*,*j*_ to CG bead *j* is analogous:

15 $${Q}_{CG,j}=\mathop{\sum }\limits_{i=1}^{{N}_{{{{{{{{\rm{cell}}}}}}}}}}{q}_{aa,i},$$

where *q*_*aa*,*i*_ is the charge of atom *i* in the Voronoi cell *j*.

Nonbonded interaction terms, particularly the Lennard-Jones *ϵ*, well depth, a parameter for each bead *j* are computed based on the solvent accessible surface area (SASA), *σ*, of the atoms within the Voronoi cell^41^. That is

16 $${\epsilon }_{j}={\epsilon }_{\max }{\left(\frac{{\sigma }_{j}^{{{{{{{{\rm{hydrophob}}}}}}}}.}}{{\sigma }_{j}^{{{{{{{{\rm{tot}}}}}}}}}}\right)}^{2},$$

where ${\sigma }_{j}^{{{{{{{{\rm{hydrophob}}}}}}}}}$ and ${\sigma }_{j}^{{{{{{{{\rm{tot}}}}}}}}}$ are the hydrophobic SASA and total SASA of the atomic domain in the Voronoi cell, respectively, and where ${\epsilon }_{\max }$ is the maximum well-depth specified by the user. This formulation improves a previous formulation of the method where all beads are defined with a fixed *ϵ* value^40^.

Finally, the last property assigned to CG beads following tessellation is the bead’s radius, which is important in properly representing the shape of the atomistic input. This is accomplished by computing the radius of gyration, *r*_gyr_, of the atomic domain with a given Voronoi cell.

Based on the above formulation, particularly equations (14) and (15), it is clear that such a CG reduction technique suffers a loss of information in low-granularity use-cases. Information on the atomistic charge or hydrophobicity profiles, for instance, are critical for multimeric biological assemblies. In previous SBCG studies, assembly stability is maintained through specific, parameterized intermonomer interactions^42^, ostensibly in the absence of detailed electrostatics and hydrophobicity information which are lost in the ~150 atoms/bead mapping.

Utilizing the topology representing the aforementioned network requires the user to specify the granularity of the model, *N*_beads_, as a free parameter. On first inspection, it might seem trivial to increase the model granularity by simply increasing the *N*_beads_ parameter, and therefore prevent loss of information as outlined above. In practice, however, the legacy implementation fails to converge for any level of granularity finer than ~40–50 atoms/bead (Supplementary Figs. 3, 4). We addressed the lack of convergence issue with modifications to the method and implemented the changes in CGBuilder in VMD, as explained below.

#### Convergence of the topology representing network

Failed convergence of the topology representing network is caused by unintended reflexive connections, latent from how neuronal states are initialized prior to optimization. In learning theory, two neurons *a* and *b* are mediated by a reflexive connection if *a* ≡ *b*. That is, if *a* and *b* are indistinguishable from the optimization procedure, meaning their stimuli and associated scoring are identical, then their relationship is reflexive.

For the topology representing network implementation herein, we found that incident reflexive connections led to undefined behavior resulting in failed convergence. Specifically, in determining a graph representing a Delaunay triangulation via a winner-take all selection rule, network behavior in the presence of a tie is undefined. If **w**_*i*_ = **w**_*j*_, the inequality

17 $$||{{{{{{{\bf{v}}}}}}}}-{{{{{{{{\bf{w}}}}}}}}}_{i}||\le||{{{{{{{\bf{v}}}}}}}}-{{{{{{{{\bf{w}}}}}}}}}_{j}||$$

in step (iii) of algorithm 2 will evaluate identically for both *i* and *j*, leading to identical adaptation of **w**_*i*_ and **w**_*j*_ in the following step. Most critically, steps (v)–(vii) of algorithm 2 will determine *C*_*ij*_ to be the strongest synapse, refresh its associated age *t*_*ij*_ to zero, age every other connection made to *i*, then remove all other connections to *i* from **C** that exceed the age threshold.

Initialization of the network involves the instancing of one neuron per each *N*_beads_. The input patterns are drawn from the atomistic structure^39^, and Cartesian coordinates are pseudo-randomly assigned from the input to initialize the weights ${\{{{{{{{{\bf{w}}}}}}}}\}}_{i=1}^{{N}_{{{{{{{{\rm{beads}}}}}}}}}}$ of neurons. During optimization, the weights are iteratively updated toward unique domains of input atoms (Movie M1) to which they are more proximal than any other (algorithm 2). For two neurons initialized with identical states, optimization forces them to identical final states. Only one bead will be assigned the properties of the atomic domain solved by the optimization and the remaining bead, resulting from reflexivity, remains unmapped to the input pattern with no assigned properties.

Our approach to enable higher granularity modeling enforces exclusivity among the initial states of neurons. During initialization, we maintain a record of which atoms among the input have already been utilized as an initial state. During the pseudo-random selection of initial states, the record is conferred to assert that a given state has not yet been utilized, and if it has, we pseudo-randomly select another state. By enforcing an exclusivity condition while initializing the network, the optimization can be successfully applied to high-granularity use-cases.

### Macromolecular assembly simulations

With the resulting SBCG2 parameters for each model, we proceed to construct our macromolecular assemblies. Generally, applying a monomeric model to a multimer involves transferring the SBCG2 mapping of a single monomer to each subunit in the assembly. A critically important detail at this stage is that the subunit subjected to the initial CG reduction is identical in sequence and structure to those comprising the assembly.

In the following sections, the multimeric assembly mapping procedure will be discussed. Further, with the goal of including inositol hexakisphosphate in the SBCG2 conical capsid model, we will elaborate guidelines for including CG ions and small molecules, and highlight the importance of performing counter ionization via the model’s Coulombic potential, the latter step, which is critical in the sub-nanometer model regime due to increased charge fidelity (Fig. 5). Finally, we will discuss simulation configuration; and importantly, determination of the integration time step via calculation of model bond frequencies.

#### Extension of SBCG2 to heteromultimeric assemblies

In SBCG2 modeling, CG mapping refers to the atomic domain assigned to each bead following spatial tessellation according to the topology representing the neural networks. Recall that each Voronoi cell emergent from network optimization bounds a domain of atoms, and the CG bead located at the center of mass of this domain is assigned the properties of constituent atoms. The multimeric mapping or map transfer utilizes the information of the CG mapping to locate each domain, Voronoi cell, in equivalent atomistic subunits comprising the assembly. Topology and parameters, e.g., bonds, angles, mass, and charge, derived in previous steps are copied to the new CG subunits.

For the map transfer operation to be successful, each target atomistic subunit must be identical in sequence and structure to the original structure employed for CG reduction. The necessity for equivalence manifests from the identification of atomic domains mapped to each bead. If these domains are in different spatial locations, then bonds, and angles joining them will be violated when topology and parameters are copied to the new subunit. Additionally, if differences in sequence are present, then the map transfer as implemented may fail completely, or place beads in positions not intended by the user.

We recommend performing separate CG mappings and parameterizations for unique structures, if the assembly is heterogeneous. For homomultimeric assemblies, taking care to construct a target atomistic assembly from identical subunits will bypass this problem entirely. Proprietary or in-house alignment protocols may further be employed as a solution to mapping to similar, but not equivalent, structures.

#### Coarse-grained ions and small molecules

CG flavors and force fields have different ways of treating ionic or otherwise charged species. In the present study, anionic and cationic species, chloride and sodium, were treated as groups of ions which carry either a −1 or +1 charge, respectively^43^. Given the granularity of our models, groups of five positive and negatively charged ions were clumped together. The latter choice was made according to the largest bead, by mass, in our SBCG2 protein topology. In our testing, the inclusion of ionic species with vastly different mass than that of protein beads caused numerical instability when attempting to utilize large integration time steps.

Our SBCG2 conical capsid model includes an additional, small molecule species: inositol hexakisphosphate, or IP6 (Supplementary Fig. 5a). IP6 is a highly charged molecule, at −12 *e*, and a known assembly co-factor for HIV-1 capsids^46,47^. In our model system, IP6 is treated as a single bead of radius 5 Å (Supplementary Fig. 5b). SBCG2 IP6 was assigned a charge of −12 *e*, and 253 were placed corresponding to the 253 capsomers comprising the conical capsid (Supplementary Fig. 5c). In atomistic HIV-1 CA hexamers and pentamers, IP6 resides approximately perpendicular to the Arg 18 ring situated at the central pore^69^. We utilized this information to place SBCG2 IP6 beads in our model (Supplementary Fig. 5c).

#### Counter ionization via 3D Coulombic potential

As we have pointed out, sub-nanometer SBCG2 models have high charge fidelity, and it is, therefore, necessary to balance the charges of the initial model with counter ions, similar to the preparation of an atomistic model. To this end, we employ a Coulombic grid potential calculation available in VMD^53^ named CIonize. In a discretized 3D grid, CIonize computes a Coulombic potential iteratively after successive placements of ions. Interestingly, and perhaps serving as an additional validation of the detailed charge of our HIV-1 CA model, Coulombic potential calculations placed sodium and chloride in equivalent positions to where these ions are known to reside in atomistic resolution structures^49^ (Supplementary Fig. 8).

With charges balanced, and other considerations addressed, such as the inclusion of small molecules or cofactors, we now turn our attention to configuring SBCG2 molecular dynamics simulations.

### Simulation parameters: temperature control, time step selection, long-range electrostatics

In the present study, we employ the NAMD3 molecular dynamics engine^45^ for all simulations, for both optimization of parameters (Figs. 6, 7) and production simulations of our multimeric assemblies, i.e., the HIV-1 capsid and cofilin-2-bound actin filaments (Figs. 1, 2, 3). As with configuring an atomistic simulation, the selection of configuration parameters is a critical step in ensuring the physical realism of the resulting MD ensemble. Here, we place particular emphasis on temperature control, integration time step, electrostatic evaluation, and the cut-off scheme, which, in a sub-nanometer context, have additional importance compared to low-granularity SBCG models.

#### Integration time step

Among the most fundamental choices when configuring a molecular simulation is the value of the integration time step. In most circumstances, choosing an integration time step—and thus establishing the temporal resolution—is motivated by the scale of the system, atomic or otherwise. For instance, an atomistic simulation of a protein might employ a 1–2 femtosecond (fs) time step, small enough to capture vibrational modes of a bond to hydrogen (~10 fs). In practice, bonds to hydrogen may be constrained and access to larger or multi-timescale integration steps becomes possible. This confers better computational performance, increasing sampling and broadening the temporal resolution of the ensemble to capture collective, large-scale molecular motions.

In SBCG modeling, and particularly sub-nanometer SBCG2 modeling, the selection of the time step is determined based on two factors: the masses of the CG beads comprising the model, and the force constants, *K*_*b*_, employed in the bonded potential energy terms. Because SBCG2 does not follow a mapping scheme a priori, but rather computes a mapping through neural network optimization, time step selection depends on granularity, more specifically the resulting SBCG2 bead masses, and is motivated by evaluating vibrational frequencies in the model.

For a bond *i*, vibrational frequency *ν*_*i*_ in units of Hertz is computed according to

18 $${\nu }_{i}=\frac{1}{2\pi }\sqrt{\frac{{K}_{b,i}}{{\mu }_{i}}},$$

where *K*_*b*,*i*_ is the bonded force constant and *μ*_*i*_ is the reduced mass of the two beads involved in the bond

19 $${\mu }_{i}=\frac{{m}_{1}\times {m}_{2}}{{m}_{1}+{m}_{2}}.$$

Following the evaluation of vibrational frequency for all bonds comprising the SBCG2 topology, the time step *τ* is then taken from the set of all frequencies $\nu={\{{\nu }_{i}\}}_{i=1}^{{N}_{{{{{{{{\rm{bonds}}}}}}}}}}$ as

20 $$\tau=\frac{1}{\max \nu }.$$

That is, we compute vibrational frequencies for the complete topology and choose a time step based on the fastest vibration, i.e., the smallest oscillation period, present. Because the bonded force constants are optimized during iterative refinement, we recommend first evaluating equation (20) using the initial parameter set yielded by Boltzmann inversion (eq. (5)) of the atomistic trajectory, then re-evaluating following iterative optimization. Far-exceeding the fastest vibrational frequencies with the selected integration time step leads to numerical instability.

#### Temperature control

For all SBCG2 simulations, we sample constant temperature (NVT) ensembles with temperature control via Langevin dynamics. The latter controls temperature by coupling the particles in the system to a dissipative background force and a randomly fluctuating force. Specifically, for a particle with mass *m* and position **x**, subjected to dissipative force **f**(**x**) = − ∇ *U*(**x**), its motion is governed by equation^70^

21 $$\frac{d{{{{{{{\bf{x}}}}}}}}}{dt}=\frac{1}{m\gamma }{{{{{{{\bf{f}}}}}}}}({{{{{{{\bf{x}}}}}}}})+{{{{{{{\bf{R}}}}}}}}(t),$$

where **R** is a zero-mean, Gaussian random process^71^ such that

22 $$\langle R(t)\rangle=0\,\,{{\mbox{and}}}\,\,\langle {{{{{{{\bf{R}}}}}}}}(t){{{{{{{\bf{R}}}}}}}}({t}^{{\prime} })\rangle=\left(2\frac{{k}_{B}T}{m\gamma }\right)\delta (t-{t}^{{\prime} }).$$

Importantly, the coefficient *γ*, in units of inverse time, is a user-specified parameter that controls the strength of thermal coupling; this is also referred to as a friction term. In NAMD’s stochastic formulation of Langevin dynamics^45,71^, the dissipative and fluctuating force terms in equation (21) are added to the Newtonian equations of motion to achieve thermal coupling and, thus, temperature control. Importantly, the choice of the Langevin *γ* term has special significance to the dynamical evolution of the molecular system^72^.

Temperature control in the Langevin framework relies on several considerations, the most principal of which is the intended dynamical regime. In molecular dynamics, momentum is conserved and the inertial effects of particles are significant. In Langevin dynamics, dampening of velocities, and thus momentum, through coupling to an external thermal reservoir—introducing a stochastic differential equation to Newton’s equations of motions^73^—allows temperature control. Increasing the *γ* coupling parameter, the system tends toward the overdamped limit^72^, where inertial effects are diminished and Brownian dynamics begin to dominate. In the Brownian dynamics regime, momentum is not conserved^72^; particles comprising the system feel a random force and a drag force, or friction, relative to a constant background (eq. (21)), and thus their motions become Brownian^72,74^.

In several CG modeling contexts, we note the reported use of *γ* coefficients in excess of 10−100 ps^−1^, whereas, in atomistic molecular dynamics contexts, *γ* is typically held between 0.5−2.0 ps^−1^. It is worth noting that overdampening is one method of achieving numerical stability during simulation, granting access to larger integration time steps. We caution the reader against indiscriminately increasing their friction coefficient to dampen velocities, unless they are aware of the dynamic consequences. For instance, performing self-assembly simulations is one exemplary justification for overdampening and accessing a larger integration time step.

In our systems of the HIV-1 SBCG2 conical capsid as well the cofilin-2-bound actin filaments, we employ a *γ* of 2.0 ps^−1^, primarily to model, implicitly, the viscosity of water. Throughout testing, we observed that we could make our time step arbitrarily large by increasing *γ* indiscriminately. Achieving a large time step is desirable only from the vantage of computational performance. If increased sampling efficiency comes at the expense of the intended dynamical regime, or predictive capability, then we argue that this is not a worthwhile exchange. For SBCG2 molecular dynamics, a *γ* between 0.5−2.0 ps^−1^, in concert with an appropriate dielectric, will productively introduce some of the macroscopic effects of solvent, namely viscosity and charge screening.

#### Long-range electrostatics via particle mesh Ewald (PME)

An additional, important consideration for the simulation of sub-nanometer SBCG2 models is the treatment of long-range electrostatics. One oft-utilized technique in molecular simulation is the particle mesh Ewald (PME) approach^48,75^. In PME electrostatic evaluation, charges are interpolated on a discrete grid, or mesh, to compute the electrostatic potential. This method is parallelizable and has been described in detail, and the specific implementation employed in the NAMD molecular dynamics engine has similarly been described^45,71^. We employ PME to treat long-range electrostatics in sub-nanometer SBCG2 simulations.

Utilizing PME in MD simulations confers detailed electrostatic treatment at the expense of performance. In our testing of the HIV-1 conical capsid, PME reduces the performance of our simulations by an approximate factor of four compared to truncated dynamics without any long-range electrostatic component (Fig. 1d, e). The resolution, in Å, of the grid to which charges are cast, is a free parameter. We have found that a grid resolution of 2 Å with a corresponding interpolation order of eight allows us to recover some of the lost performance, without sacrificing accuracy or numerical stability. The selection of grid resolution and interpolation order, are use-case-specific considerations. Further, the choice of electrostatic cut-off distances is an associated dependency in treating long-range electrostatics, which is discussed in the following section.

For the cofilin-2-bound actin filaments, we find that PME electrostatic evaluation leads to a more significant reduction in performance (Fig. 2d, e) compared with truncated dynamics. The reason for this is related to the spatial decomposition of the filamentous systems, which have significantly large ratios of length to cross-sectional area. This point is notable, since SBCG2 modeling pushes molecular simulation to considerable size scales. We are motivated to address the latter in future work.

#### Nonbonded interaction cutoffs

Related to establishing parameters for the PME grid is the assertion of cut-off distances. In the NAMD molecular dynamics engine, the cut-off scheme is described with three parameters: a cut-off distance, beyond which the long-range potential is truncated; a switching distance (if switching is enabled in the configuration), which specifies the distance beyond which a splitting function is employed; and the pair list distance, which determines the maximum considerable pair distance between any two particles.

Fundamentally, cut-off distances should be larger than the longest bond term in the CG topology; however, increasing electrostatic cut-off distance leads to larger computational expense, since more bead pairs in the pair list necessitate more evaluations. Utilizing the longest bonded distance in the topology as a lower bound, we employ an upper bound based on the interfacial distances in our biomolecular assembly. This approach is equivalent to an approach used to select cut-off distances in a previous SBCG study of capsids^42^.

#### GPU-accelerated SBCG2 simulations

The sampling efficiency of SBCG2 simulations benefits significantly from GPU acceleration. Typical GPU accelerators have their own dedicated memory of 8 to 24 GB as of the time of this publication. In the GPU-accelerated computing paradigm, problems that fit neatly within the memory of the graphics processor are amenable to multiple-factor speedups^76^. In contrast, problems that exceed dedicated accelerator memory lead to costly host-to-device copy operations and excessive communication overhead, which place a hard limit on attainable sampling efficiency. The design strategy of MD engines such as NAMD2^71^ is to offload only a subset of computations to the GPU, namely the evaluation of nonbonded electrostatics. While selective offloading is a flexible strategy that accommodates diverse systems on heterogeneous architectures, the biggest performance gains remain unrealized.

Recently, a fully GPU-resident MD engine NAMD3^45^ was developed, which offloads all computations to the GPU. Multimeric SBCG2 assemblies, such as the HIV-1 conical capsid presented here, represent ideal memory footprints for saturating and taking full advantage of GPU acceleration. Remarkably, with certain simulation configurations such as those employing truncated dynamics (see section Long-range electrostatics via particle mesh Ewald), we are able to achieve sampling efficiency in excess of 1 microsecond per day using NAMD3 (Fig. 1e) for the HIV-1 capsid, and greater than 3 µs per day for our three-turn filament system (Fig. 2e). Employing full electrostatic evaluation with PME for simulations of the HIV-1 capsid, we can still reach high sampling rates in excess of 300 ns per day (Fig. 1d). The latter two performance metrics represent significant speedups over CPU (Supplementary Fig. 2), or heterogeneous CPU and GPU, computation. Furthermore, our benchmark analysis shows that the performance of multimeric SBCG2 assemblies scales across multiple GPUs. Supplementary Table 1 shows benchmarks of the three-turn cofilin-2 bound actin filament system utilizing NVIDIA’s DGX A100, employing varying numbers of cores per GPU utilized. Remarkably, utilizing eight A100 GPUs with eight CPUs per GPU, yielding 64 in total, we exceed four microseconds per day simulation performance.

### Reporting summary

Further information on research design is available in the Nature Portfolio Reporting Summary linked to this article.

## Supplementary information

Supplementary Information

Description of Additional Supplementary Files

Supplementary Movie M1

Supplementary Movie M2

Supplementary Movie M3

Supplementary Movie M4

Reporting Summary

## Data Availability

Raw and processed data generated in this study as well as example scripts demonstrating new aspects of the code, have been deposited into a Zenodo repository and are freely accessible to the public at 10.5281/zenodo.7685834. Source data for all main text figures are provided with this paper as a Source Data file. The following previously published structures were used in this study: PDB 7U8K (Kraus et al., cofilin-2 bound to actin^44^), EMDB 13423 (Ni et al., IP6-bound HIV-1 CA hexamer^46^), EMDB 13422 (Ni et al., IP6-bound HIV-1 CA pentamer^46^) Source data are provided with this paper.

## Source data

Source Data

## Code availability

SBCG2 will be available as part of the CGBuilder plugin, distributed with Visual Molecular Dynamics (VMD) versions 1.9.4 and later. For users wishing to try the new method now in their existing VMD installation, a repository containing the code, with a script to handle independent loading of the new plugin, and a README describing usage, is available to the public at 10.5281/zenodo.7685834. The FSC calculation implementation in VMD will be made available as part of the 1.9.4 release of the software and can be obtained by contacting the authors.
