# Supplementary material for: Performance efficient macromolecular mechanics via sub-nanometer shape based coarse graining
Source: Nat Commun. 2023 Apr 10;14:2014. doi: 10.1038/s41467-023-37801-5 (PMC10086035; doi:10.1038/s41467-023-37801-5)
Supplement: Supplementary file 1 — Supplementary Information [file 41467_2023_37801_MOESM1_ESM.pdf]

Supplementary Information

DGX A100 640GB						
No PME				PME		
GPUs	CPUs per GPU	ns/day		GPUs	CPUs per GPU	ns/day
1	8	1497.62		1	8	481.657
**1**	**16**	**1564.61**		**1**	**16**	**496.476**
1	32	1539.68		1	32	491.068
1	64	1474.44		1	64	485.423
1	128	1327.73		1	128	442.508
2	8	2305.98		2	8	548.472
2	16	2370.77		2	16	549.247
**2**	**32**	**2433.6**		**2**	**32**	**552.919**
2	64	2265.39		2	64	532.441
4	8	3111.83		4	8	595.852
**4**	**16**	**3247.3**		**4**	**16**	**598.493**
4	32	3225.44		4	32	597.041
**8**	**8**	**4038.3**		8	8	621.336
8	16	3711.83		**8**	**16**	**621.695**

Table 1: Performance benchmarks of the 3-turn cofilin-2-bound actin filament system utilizing NVIDIA's DGX A100, with 640 GB of total memory. On the left, performance metrics are obtained for SBCG2 MD simulations with PME *off*. On the right, performance metrics obtained for SBCG2 MD simulations with PME *on*. For each number of GPUs employed, the number of CPUs providing optimal performance is shown in bold.

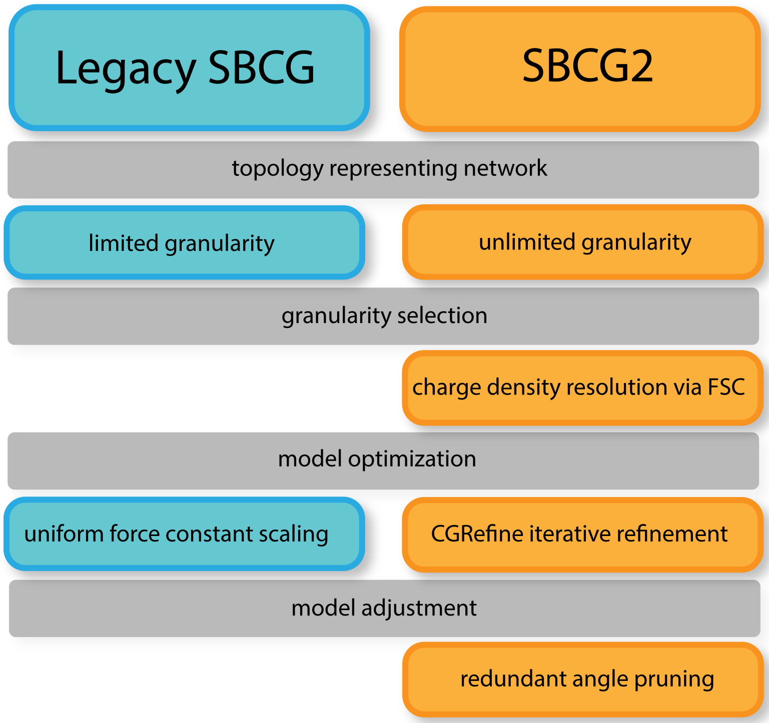

Supplementary Figure 1: Feature comparison of legacy shape based coarse graining (SBCG) vs. our new and improved SBCG2 version. Each category (gray box) represents an area where SBCG2 was modified or improved, and legacy SBCG is denoted in cyan while the new SBCG2 in orange.

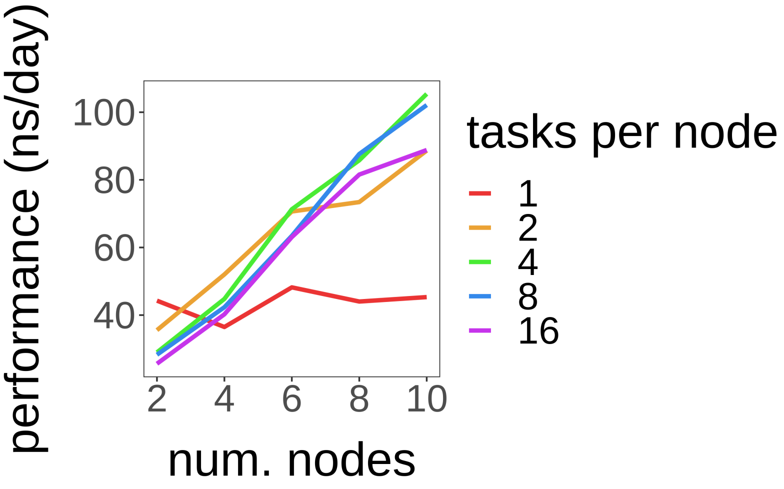

Supplementary Figure 2: Multinode CPU scaling of the SBCG2 HIV-1 conical capsid, with PME electrostatic evaluation. This scaling analyses employs no GPU acceleration, and serves as a point of comparison for the fully GPU-resident NAMD3 benchmarks presented in the main text. For each number of nodes tested, the number of tasks bound to each node was varied and colored according to the legend provided. Benchmarks reported are the mean value of the six benchmark metrics reported by NAMD3 [45] for each simulation.

a

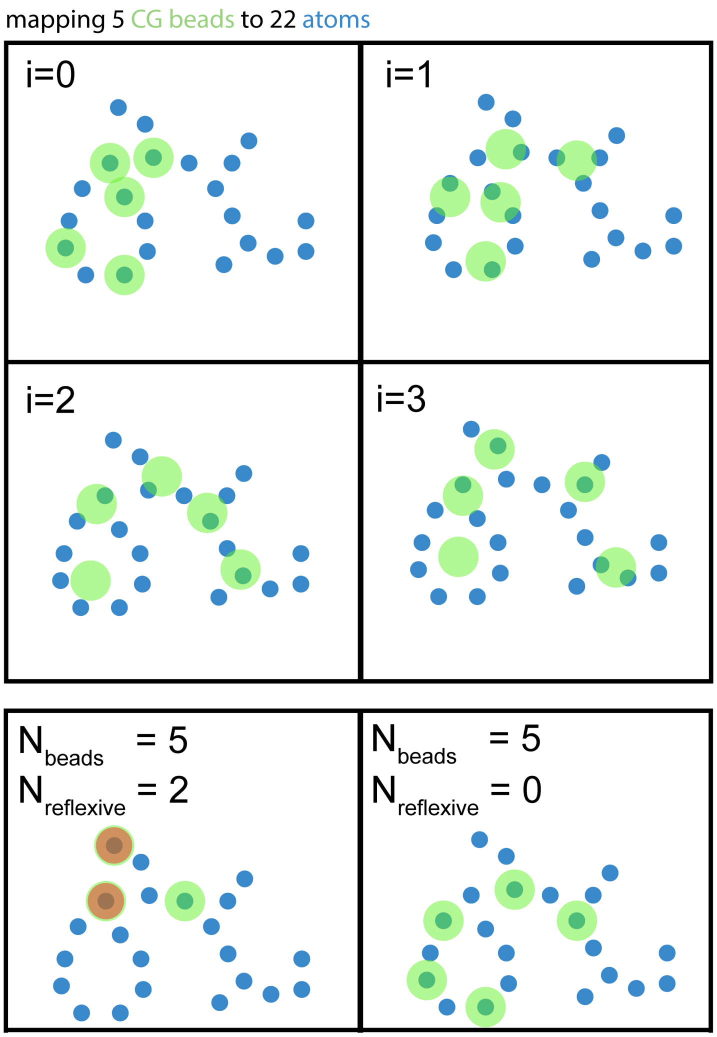

b

Supplementary Figure 3: Graphical overview of the topology representing network with a 2-D cartoon example. **a** Exemplary 2-D graphic showing the mapping of five CG beads (green) to 22 atoms (blue), over four iterations, or *learning steps*, *i*. **b** Left, example case of unintended *reflexive connections* (red), where two CG beads are initialized to the same coordinate prior to network optimization. This case results in two assignment failures. Right, the same example but where each bead and its associated weight begin at a unique starting position.

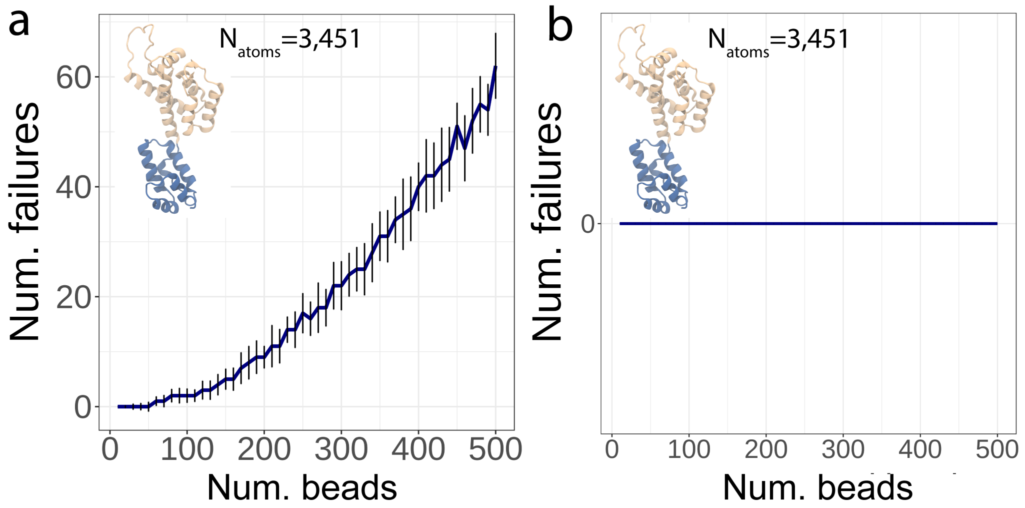

Supplementary Figure 4: Assignment failures in the Topology Representing Network (TRN) resulting from reflexivity. **a** Without an exclusivity condition among neuronal weights, runs of the TRN produce assignment failures, the latter which grows with the number of requested beads. **b** With an exclusivity condition among initializing neuronal weights, runs of the TRN produce no assignment failures across all 1,000 trials performed. To perform this analysis for both panels, the relevant code was extracted from the legacy CGBuilder plugin and written into a simulator, which for a given atomistic input pattern (in this case HIV-1 CA, shown inset) can determine how many assignment failures will manifest. The analysis performed tests over $Num_{\mathrm{CG}} \in [10, 500]$ with a stride of 10 for an input of 3,451 atoms, and each value of requested beads was simulated 20 times. The error bars denote the standard deviation over these 20 tests ($n = 20$), the latter deviation among the ensembles results from pseudo-random selection. For panel b, the exclusivity condition was enforced but otherwise an identical test was performed. As shown, our correction to the initialization of weights prevents any redundancies, and therefore assignment failures, enabling the TRN to produce models of much higher granularity.

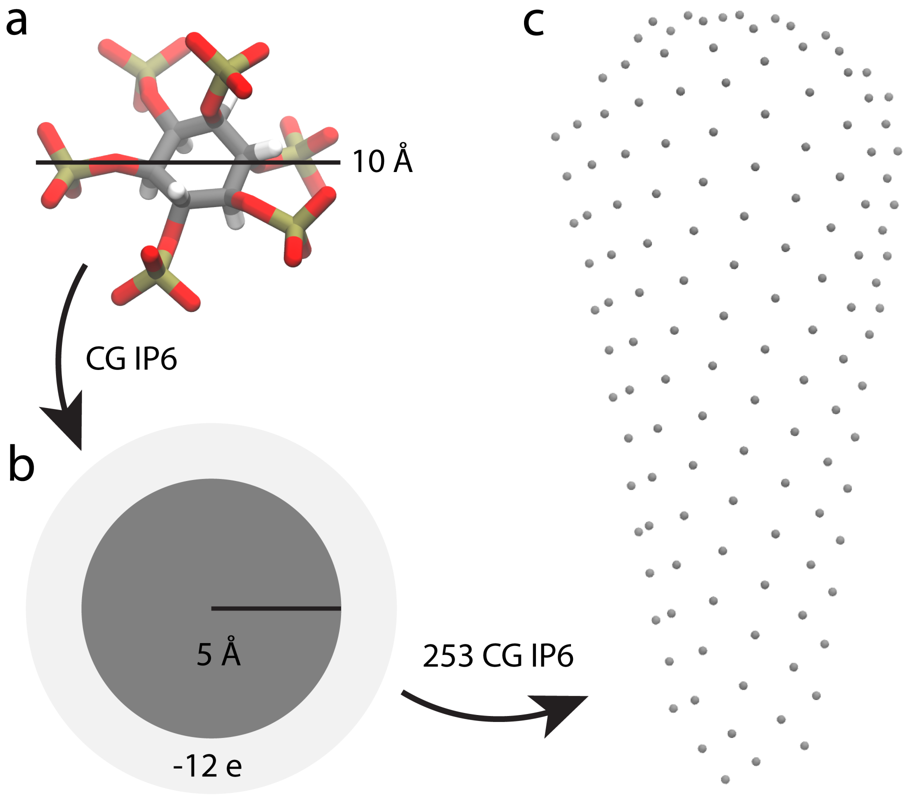

Supplementary Figure 5: Overview of the CG description and placement of inositol hexakisphosphate (IP6) assembly cofactor. **a** Atomistic IP6 model, measured with a diameter of 10 Å. **b** Single-bead CG IP6, parameterized with a diameter of 5 Å and a charge of -12 *e*. **c** Final placement of CG IP6 beads in our conical capsid model. One CG IP6 is placed at the center pore of each capsomer, yielding 253 in total. The beads are placed proximal to beads mapped to side chains of Arginine 18.

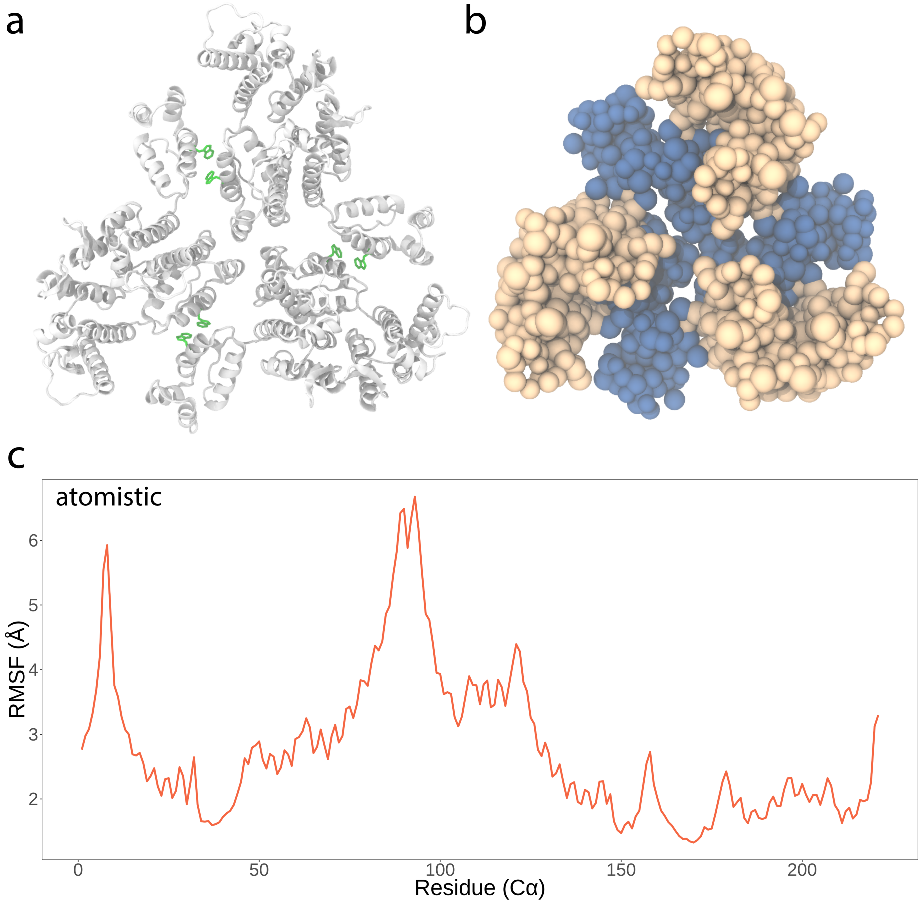

Supplementary Figure 6: HIV-1 CA trimer of dimers construct utilized in parameterization. **a** Atomistic CA trimer of dimers, simulated at 298 K for 80 ns. Protein is shown in cartoon representation. Tryptophan 184, critical for intermolecular dimer interface stability, is shown in green licorice representation. **b** Resulting SBCG2 trimer of dimers, which was simulated throughout iterative refinement (Figures 6 and 7). **c** Root-mean-square fluctuation (RMSF) of alpha Carbons of the atomistic construct in panel a.

a

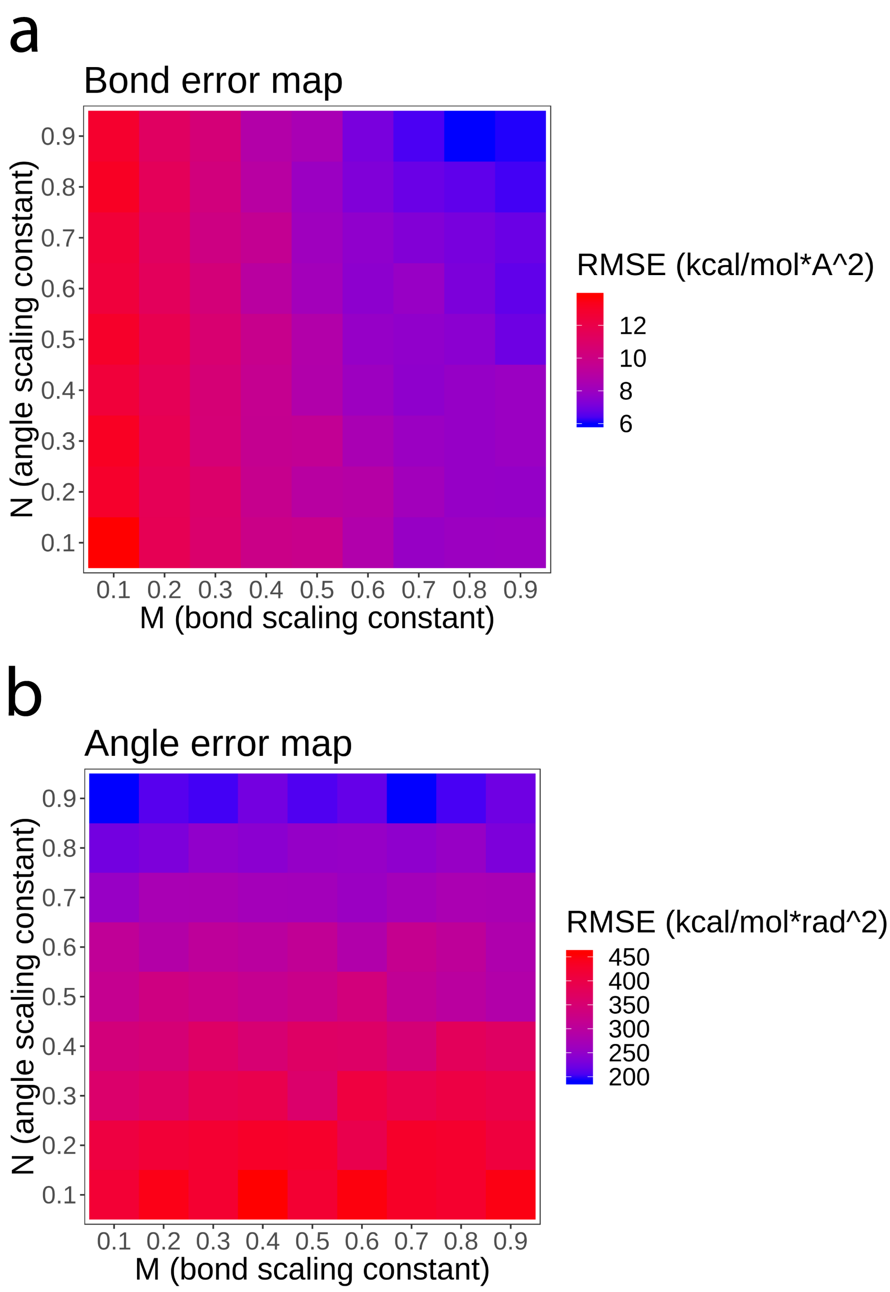

Supplementary Figure 7: Parameter sweep for identifying optimal m (bonds, **a**) and n (angles, **b**) scaling constants. The heat map shows the root-mean-square error for 81 simulations – a 9×9 grid – computed for a single refinement iteration. Blue denotes lower error and red denotes higher error, with scale bars provided.

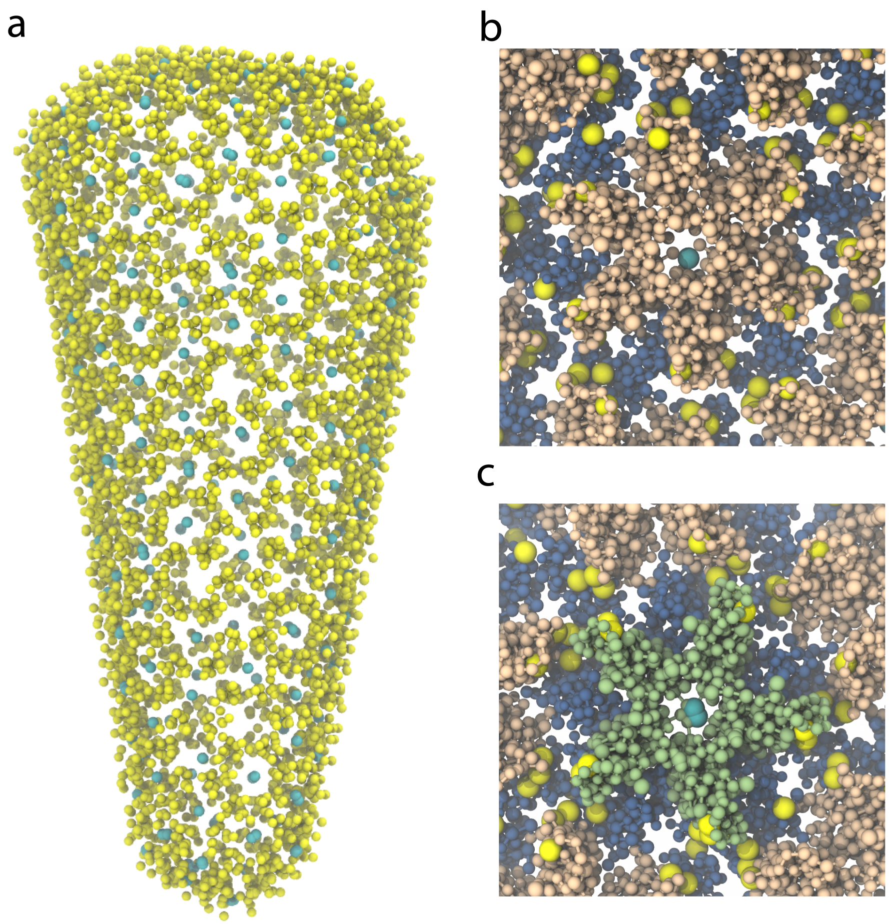

Supplementary Figure 8: Counterion placement around the SBCG2 HIV-1 conical capsid. Radii of protein and ion beads are not to scale, but selected for visual clarity. **a** Counterions of mixed nature, carrying either a positive (yellow) or negative (cyan) charge, as placed according to iterative Coulombic grid potential calculations. **b** Close-up of a CA hexamer from the conical capsid, showing counterions. **c** Close-up of a CA pentamer from the conical capsid, showing counterions.

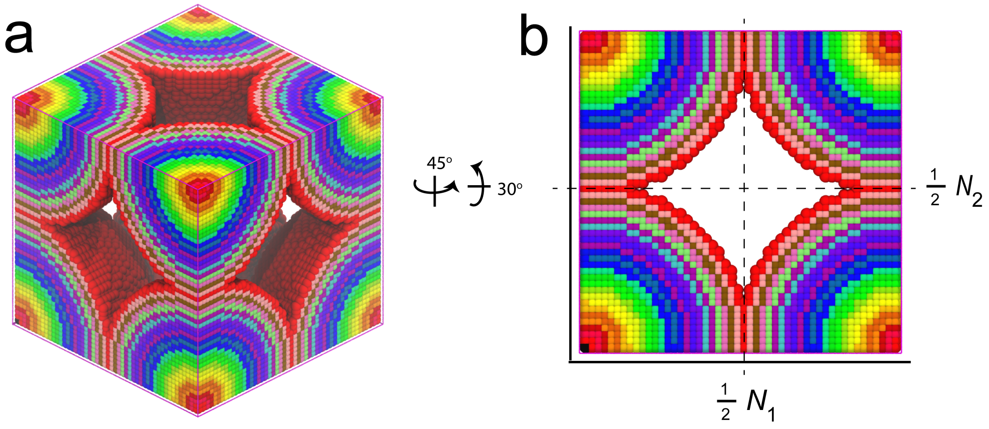

Supplementary Figure 9: Example visualization of Fourier Shells, used in measuring three dimensional Fourier Shell Correlation (FSC). In FSC analysis, the normalized correlation between complex structure factors, derived from two volumetric data sets, are compared at specific spatial intervals (eq. 2). In three dimensions, spatial bins comprise Fourier shells, with a thickness equal to one voxel width. **a** Shows a cubic grid, with voxel elements colored by radial bin. The voxel corresponding to the map origin is colored black. **b** Shows a view of the same grid, normal to the $\hat{x}$-$\hat{y}$ plane. In this illustration, the Nyquist frequencies along the $\hat{x}$ and $\hat{y}$ dimensions are annotated and shown as dashed lines. Frequencies beyond the Nyquist limit are aliased, resulting in a periodic access pattern.

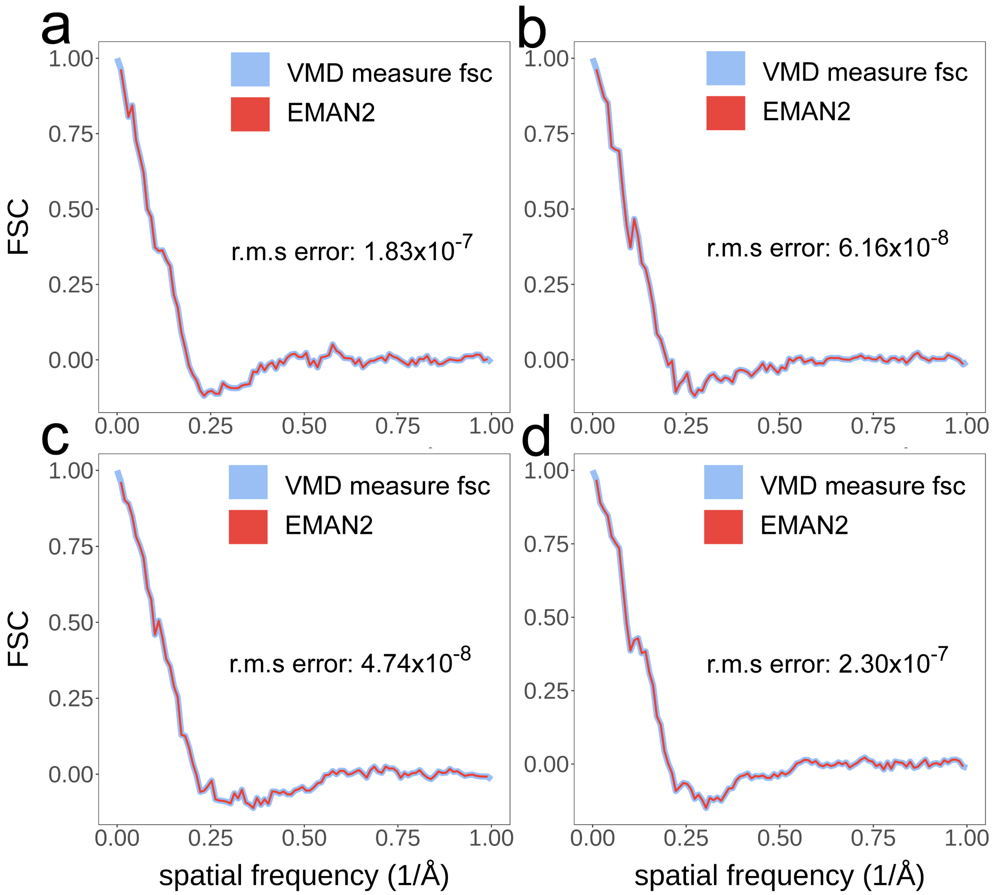

Supplementary Figure 10: Comparison of our C++ GPU-accelerated FSC implementation, *measure fsc*, vs. the widely-used EMAN2 software [56]. Each of the four plots shows our result, blue, and EMAN2's, red, for the same input densities. In the EMAN2 test case, we used UCSF Chimera [58] for resampling the SBCG2 density on the atomistic reference density. In the *measure fsc* test cases, we used our own, built-in resampling procedure. Root mean square error is provided for each plot, demonstrating that our results conform within floating point error tolerance. **a** SBCG2 HIV-1 charge density FSC, with 175 beads. **b** SBCG2 HIV-1 charge density FSC, with 180 beads. **c** SBCG2 HIV-1 charge density FSC, with 200 beads. **d** SBCG2 HIV-1 charge density FSC, with 210 beads.

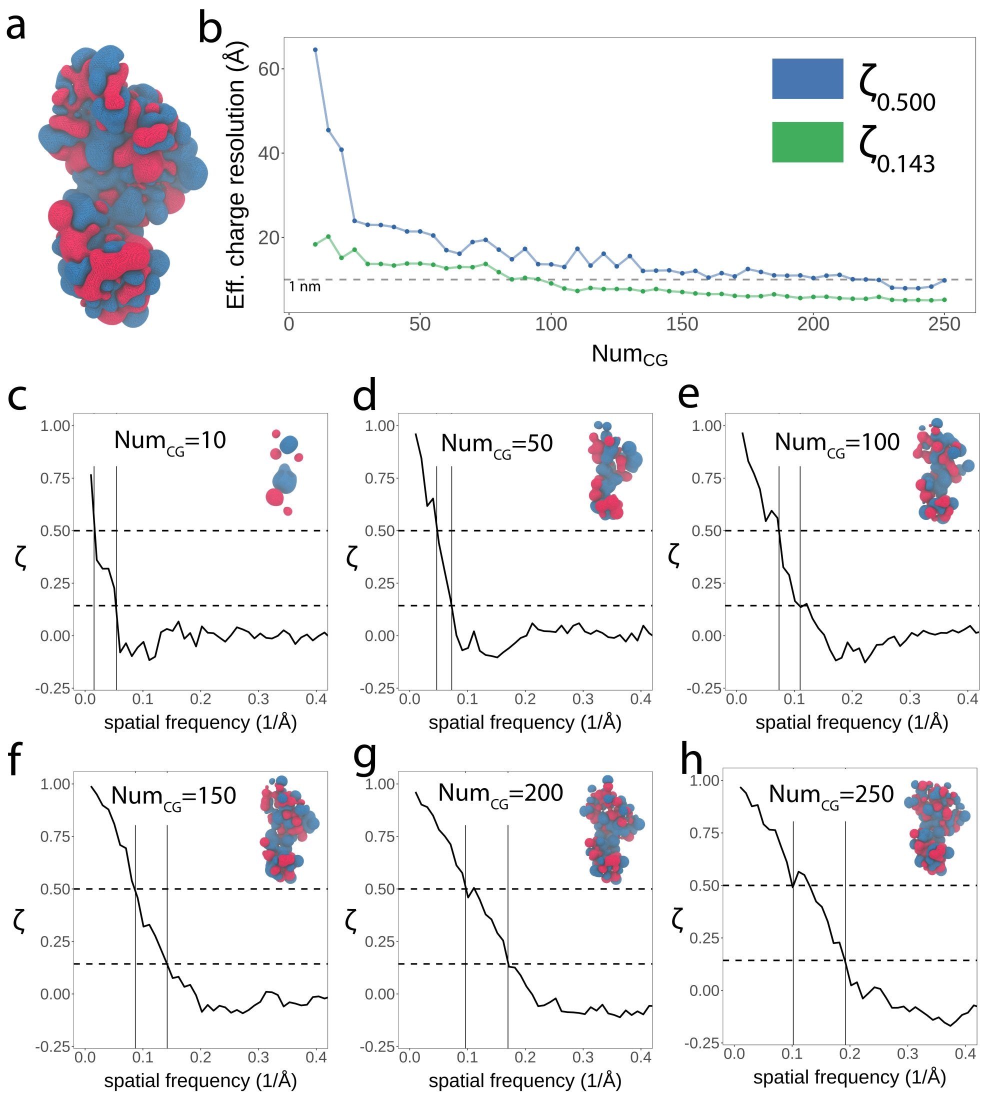

Supplementary Figure 11: FSC analysis of CG granularity vs. effective charge density resolution. **a** Charge density of the all-atom reference structure of HIV-1 CA. Regions of positive and negative charge density are colored blue and red, respectively. **b** Effective charge density resolutions for models $\mathrm{Num_{CG}} \in [10, 250]$, plotted with two metrics: $\zeta_{0.143}$ and $\zeta_{0.500}$, green and blue, respectively. The dotted gray line represents a resolution of 1 nm. **c-h** Select charge density isosurfaces and FSC plots, $\mathrm{Num_{CG}} \in \{10, 50, 100, 150, 200, \text{ and } 250\}$. Charge densities are shown with identical isovalues and coloring to the density in panel a. Dashed horizontal lines denote each of the metrics employed, $\zeta_{0.143}$ and $\zeta_{0.500}$. Vertical solid lines in the plots denote the determination, via point of intersection, of effective resolution with $\zeta_{0.143}$ and $\zeta_{0.500}$ metrics.

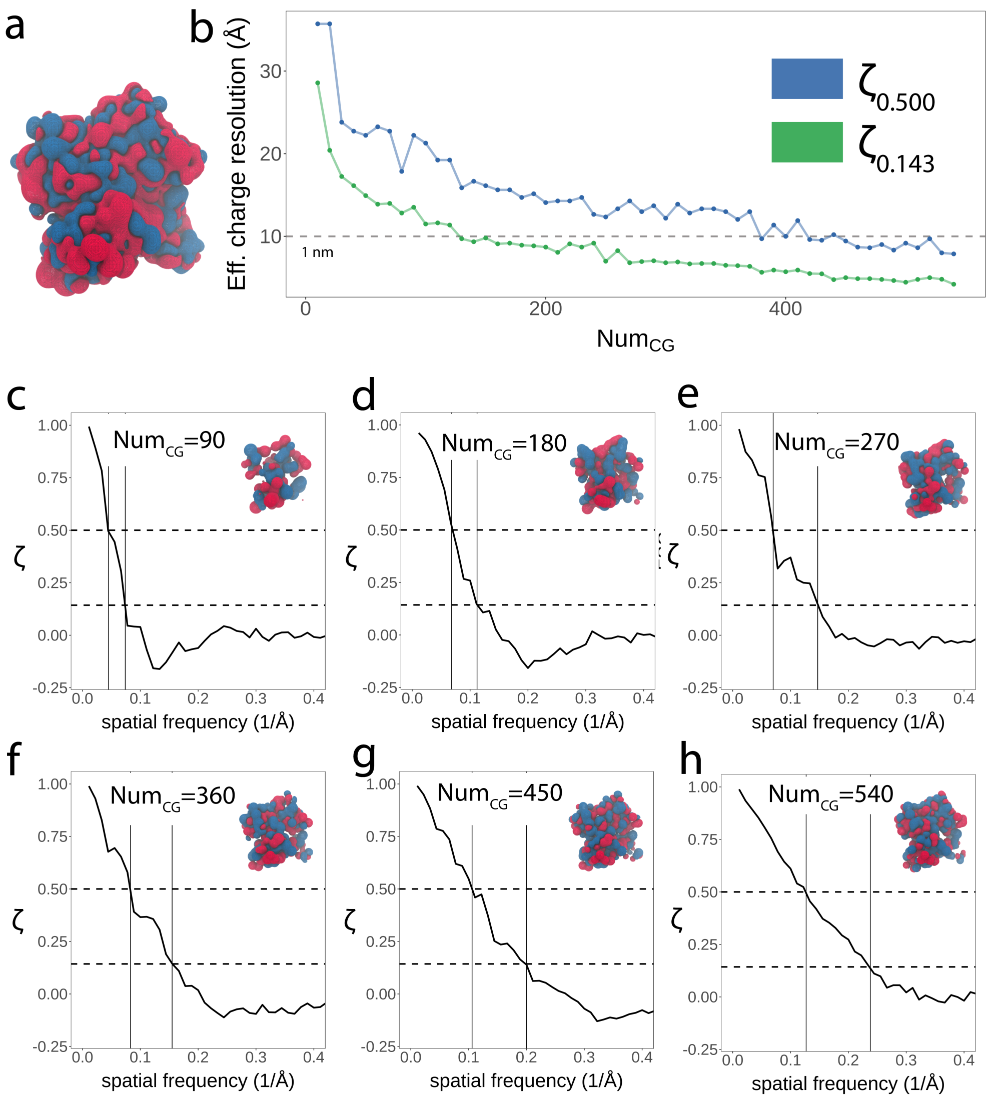

Supplementary Figure 12: FSC analysis of CG granularity vs. effective charge density resolution. **a** Charge density of the all-atom reference structure of actin. Regions of positive and negative charge density are colored blue and red, respectively. **b** Effective charge density resolutions for models $\text{Num}_{\text{CG}} \in [10, 540]$, plotted with two metrics: $\zeta_{0.143}$ and $\zeta_{0.500}$, green and blue, respectively. The dotted gray line represents a resolution of 1 nm. **c-h** Select charge density isosurfaces and FSC plots. Charge densities are shown with identical isovalues and coloring to the density in panel a. Dashed horizontal lines denote each of the metrics employed, $\zeta_{0.143}$ and $\zeta_{0.500}$. Vertical solid lines in the plots denote the determination, via point of intersection, of effective resolution with $\zeta_{0.143}$ and $\zeta_{0.500}$ metrics.

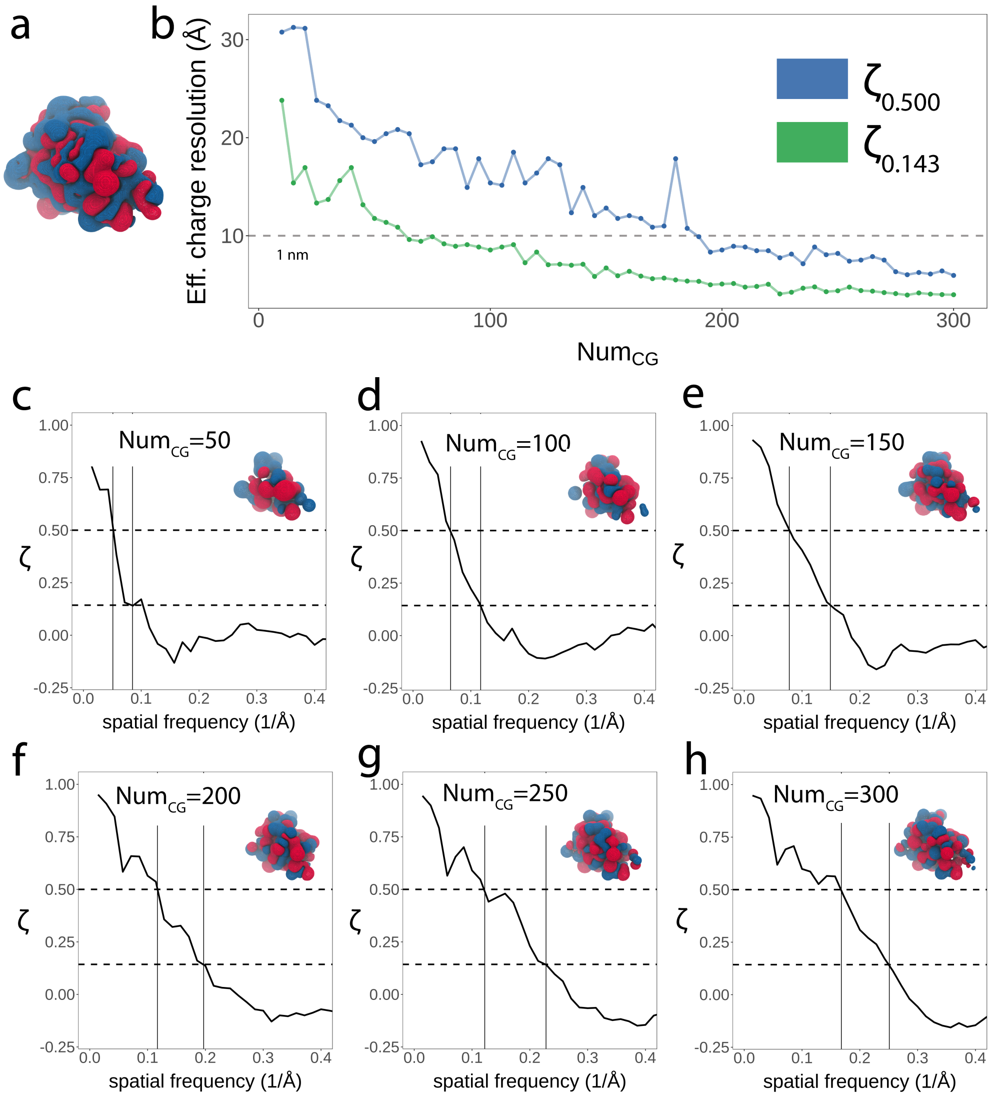

Supplementary Figure 13: FSC analysis of CG granularity vs. effective charge density resolution. **a** Charge density of the all-atom reference structure of cofilin-2. Regions of positive and negative charge density are colored blue and red, respectively. **b** Effective charge density resolutions for models $\text{Num}_{\text{CG}} \in [10, 300]$, plotted with two metrics: $\zeta_{0.143}$ and $\zeta_{0.500}$, green and blue, respectively. The dotted gray line represents a resolution of 1 nm. **c-h** Select charge density isosurfaces and FSC plots. Charge densities are shown with identical isovalues and coloring to the density in panel a. Dashed horizontal lines denote each of the metrics employed, $\zeta_{0.143}$ and $\zeta_{0.500}$. Vertical solid lines in the plots denote the determination, via point of intersection, of effective resolution with $\zeta_{0.143}$ and $\zeta_{0.500}$ metrics.

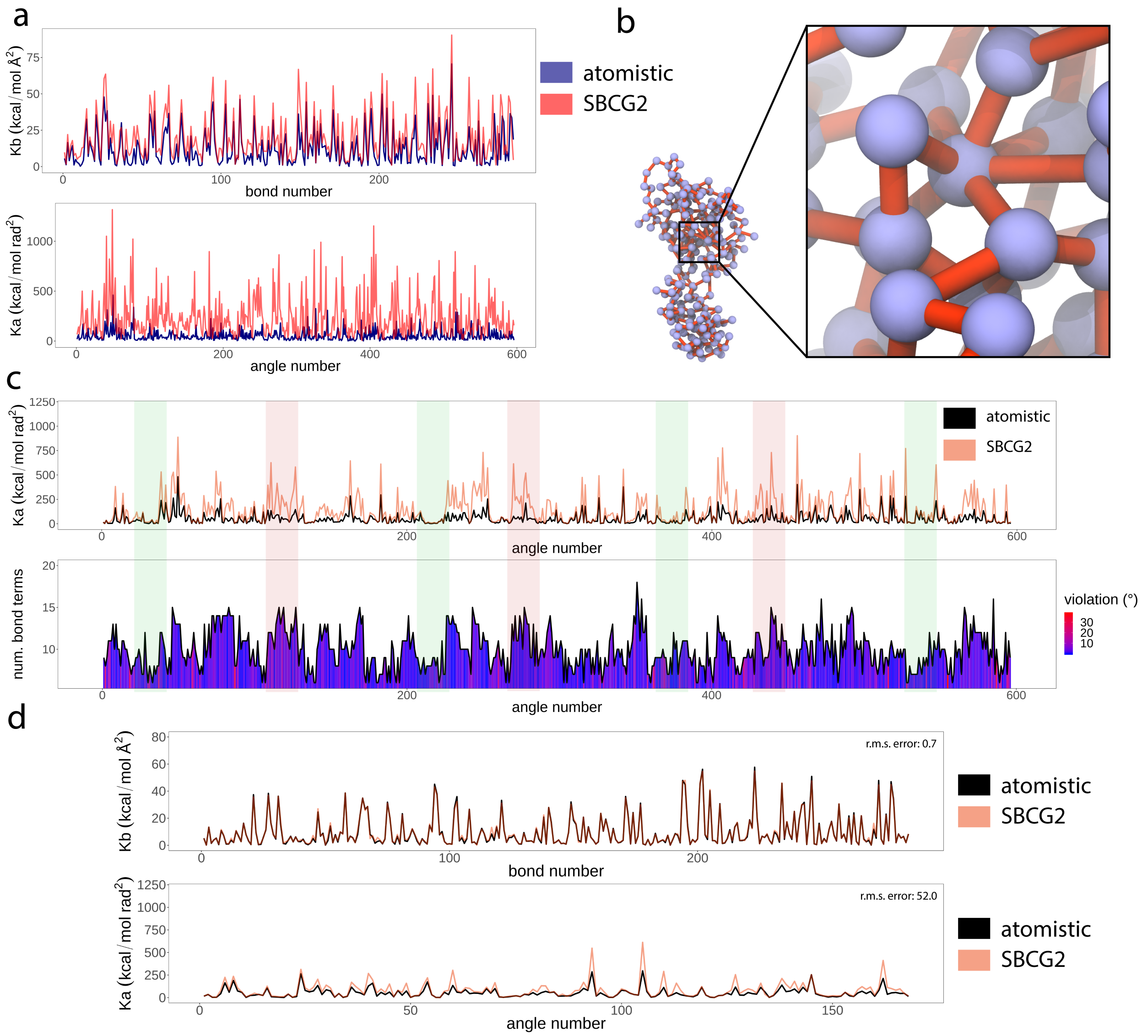

Supplementary Figure 14: SBCG2 HIV-1 CA parameter optimization results. **a** Initial parameter set (red) derived from Boltzmann inversion of the all-atom trajectory (blue). Bond and angle (top and bottom, respectively) indices are given along the $\hat{x}$-axis, and the corresponding force constants are plotted along the $\hat{y}$-axis. This plot demonstrates the poor quality of the initial fit, and motivates iterative refinement. **b** Example of a highly connected bead in the SBCG2 CA topology. **c** Analysis of angle parameters based on their bonded connectivity. The top panel shows angle parameters, converged to an unphysical state (orange), after many iterations of refinement (Movie **??**). The bottom panel is aligned to the top and shows a trace of bonded connectivity, per angle parameter (for beads involved in the angle, all bonds connected to them are summed). The area under the latter trace is colored by violation, i.e., deviation of the angle value from the all-atom reference value. Regions of low connectivity correspond to good fit, shaded green. Regions of high connectivity correspond to bad fit, shaded red. **d** Converged parameter refinement following the pruning of redundant angle parameters. Final root-mean-square error (RMSE) values are shown.

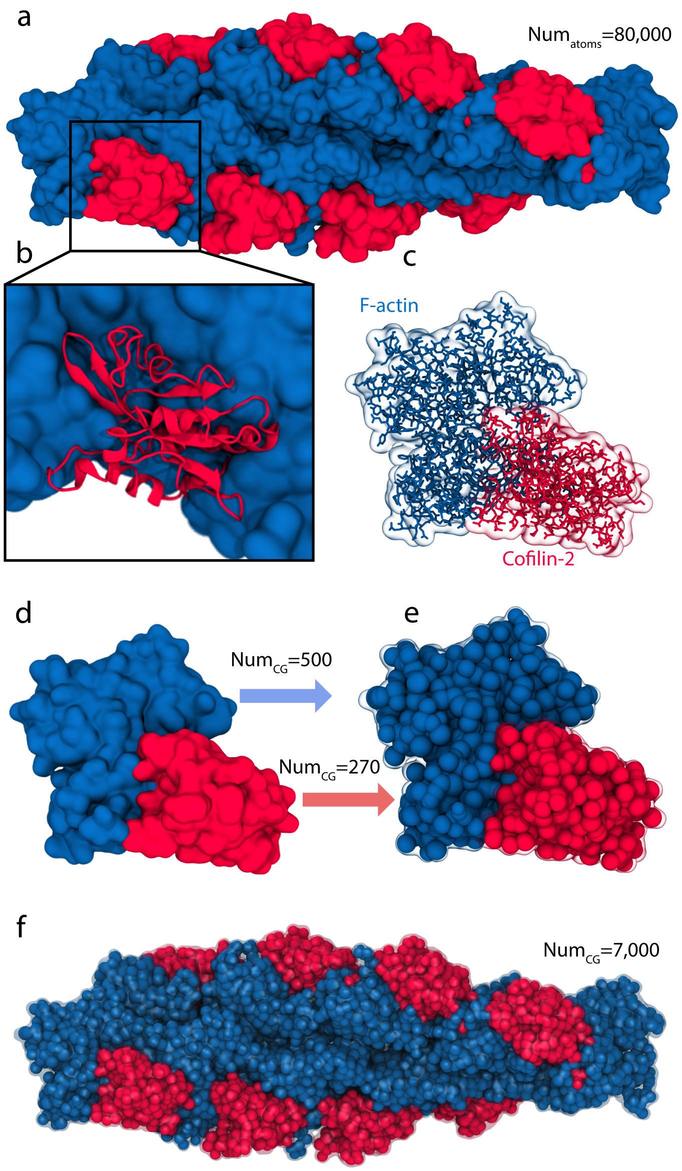

Supplementary Figure 15: Heterogeneous multimeric assembly, cofilin-2 on actin filaments. **a** All-atom surface representation of the cofilin-2 and F-actin system. **b** Zoomed-in, secondary structure view of a cofilin-2 monomer in complex with actin, the latter shown in surface representation. **c** Atomistic view, without hydrogens, of each domain, cofilin-2 and F-actin, subjected to sub-nanometer shape based coarse graining. Molecular surfaces are shown transparently. **d,e** Atomistic surfaces, left, and the sub-nanometer SBCG2 models resulting from two independent calculations. Cofilin-2 modeling employed $\mathrm{Num_{CG}} = 270$ and F-actin modeling employed $\mathrm{Num_{CG}} = 500$, corresponding to approx. 11 atoms per bead for each protein domain. **f** Resulting multimeric SBCG2 cofilin-2 actin system after performing the mapping operation. The atomistic molecular surface is also shown transparently, demonstrating the quality of the resulting SBCG2 topology
