# Supplementary material for: Performance efficient macromolecular mechanics via sub-nanometer shape based coarse graining
Source: Nat Commun. 2023 Apr 10;14:2014. doi: 10.1038/s41467-023-37801-5 (PMC10086035; doi:10.1038/s41467-023-37801-5)
Supplement: Supplementary file 2 — Description of Additional Supplementary Files [file 41467_2023_37801_MOESM2_ESM.pdf]

Description of Additional Supplementary Files

Supplementary Movie 1 – Animation of adaptation via the TRN

Supplementary Movie 2 – Animation of parameter fitting

Supplementary Movie 3 – Movie of 225 ns of SBCG2 HIV-1 conical capsid simulation

Supplementary Movie 4 – Movie of SBCG2 HIV-1 conical capsid subjected to nano-indentation
